# An assay for determining the susceptibility of *Salmonella* isolates to commercial and household biocides

**DOI:** 10.1371/journal.pone.0209072

**Published:** 2018-12-20

**Authors:** Shaheen B. Humayoun, Lari M. Hiott, Sushim K. Gupta, John B. Barrett, Tiffanie A. Woodley, John J. Johnston, Charlene R. Jackson, Jonathan G. Frye

**Affiliations:** 1 Bacterial Epidemiology and Antimicrobial Resistance Research Unit, United States Department of Agriculture, Agricultural Research Service, United States National Poultry Research Center, Athens, GA, United States of America; 2 United States Department of Agriculture, Food Safety and Inspection Service, Fort Collins, CO, United States of America; The University of Sydney, AUSTRALIA

## Abstract

Poultry and meat products contaminated with *Salmonella enterica* are a major cause of foodborne illness in the United States. The food industries use a wide variety of antimicrobial interventions to reduce bacterial contamination. However, little is known about *Salmonella* susceptibility to these compounds and some studies have shown a concerning link between biocide resistance and antibiotic resistance. To investigate this, a 96 well panel of 17 common household and commercially used biocides was designed to determine the minimum inhibitory concentrations (MIC) of these compounds for *Salmonella*. The panel contained two-fold serial dilutions of chemicals including Dodecyltrimethylammonium chloride (DC), Benzalkonium chloride (BKC), Cetylpyridinium chloride (CPC), Hexadecyltrimethylammonium bromide (HB), Hexadecyltrimethylammonium chloride (HC), Acetic acid (AA), Lactic acid (LA), Citric acid (CA), Peroxyacetic acid (PXA), Acidified sodium chlorite (ASC), Sodium hypochlorite (SHB), 1,3 dibromo, 5,5 dimethylhydantoin (DBH), Chlorhexidine (CHX), Sodium metasilicate (SM), Trisodium phosphate (TSP), Arsenite (ARI), and Arsenate (ARA). The assay was used to test the susceptibility of 88 multidrug resistant (MDR) *Salmonella* isolates from animal sources. Bacteria are defined as multidrug resistant (MDR) if it exhibited non-susceptibility to at least one agent in three or more antimicrobial categories. The concentration of biocide at which ≥50% of the isolates could not grow was designated as the minimum inhibitory concentration or MIC_50_ and was used as the breakpoint in this study. The MIC_50_ (μg ml^-1^) for the tested MDR *Salmonella* was 256 for DC, 40 for BKC, 80 for CPC. HB and HC, 1,640 for AA, 5664 for LA, 3,156 for CA, 880 for PXA, 320 for ASC, 3.0 for CHX, 1,248 for DBH, 3,152 (6%) for SHB, 60,320 for SM, 37,712 for TSP, 56 for ARI and 832 for ARA. A few isolates were not susceptible at the MIC_50_ breakpoint to some chemicals indicating possible resistance. Isolates with MICs of two 2-fold dilutions above the MIC_50_ were considered resistant. Biocides for which resistant isolates were detected included CPC (n = 1 isolate), HB (1), CA (18), ASC (7), CHX (22), ARA (16), and ARI (4). There was no correlation detected between the biocide susceptibility of *Salmonella* isolates and antibiotic resistance. This assay can determine the MICs of bacteria to 17 biocides in a single test and will be useful in evaluating the efficacy of biocides and to detect the development of resistance to them.

## Introduction

*Salmonella enterica* is highly diverse, including six subspecies and more than 2,500 serovars [1,2,3,4]. All serovars except a few which cause typhoid fever are referred to as non-typhoidal *Salmonella* (NTS). The main niche of NTS is the gastrointestinal tract of humans and farm animals such as swine, cattle, and poultry, but is also found in the intestinal tract of wild birds, reptiles, and insects surviving harsh environments [5]. The major source of *Salmonella* infections in the human population is through the consumption of contaminated food such as meat, eggs, dairy products, and fresh produce [6,7,8]. According to the Centers for Disease Control and Prevention (CDC), *Salmonella* causes more than one million human infections resulting in approximately 23,000 hospitalizations and 450 deaths in the United States each year [4,9,10]. The organism also causes economic and social impact estimated to cost an average of $4 billion dollars each year [11,12]. The cause of outbreaks in many cases is through cross-contamination of food with *Salmonella*. To reduce the risk of outbreaks, there is a need to use proper hygienic measures and effective disinfection to prevent *Salmonella* transmission to humans [13,14,15].

Biocides play an important role in controlling pathogenic bacteria and have been used for centuries as antiseptics, disinfectants, decontaminants, and sterilants in health care facilities, animal farms, food and meat processing industries, in household cleaning products, and as preservatives in pharmaceuticals, cosmetics, textiles, laundry detergents, and the food industries [16,17,18]. In the United State, biocides are regulated by the Environmental Protection Agency (EPA) and are referred as antimicrobial pesticides [19]. There are currently more than 4,000 antimicrobial products are registered with EPA containing about 275 different active ingredients and approximately one billion dollars spent each year on different types of antimicrobial products [20].

With proper use, biocides are an essential part of public health; however, misuse such as frequent exposure to sub-lethal concentrations of biocides could result in the development of resistance in bacteria to biocides and also to antibiotics [21,22,23,24,25]. The use of sub-inhibitory concentrations of biocides can also select for strains that are tolerant to these chemicals [26]. This development of resistance to biocides could render them ineffective and lead to bacterial contamination of food and reduced food safety.

Biocides and antibiotics have different mechanisms of action. Biocides have a broader spectrum of activity and act on multiple target sites altering various cellular mechanisms of bacteria; therefore, mutation within a single target is unlikely to result in biocide resistance. Alternatively, antibiotics have a high level of target specificity and antibiotic resistance is often associated with mutations in specific genes or horizontal acquisition of a foreign gene encoding a resistance mechanism [27]. Despite the differences in the mode of action of biocides and antibiotics, there are also some similarities such as the uptake through the bacterial envelope by passive diffusion, effect on membrane integrity and morphology, and the effect on bacterial metabolism [18,28]. Additionally, genes encoding biocide resistance are sometimes found on horizontally transferred mobile genetic elements that also carry antibiotic resistance genes [29,30]. Because the possibility of cross-resistance between antibiotics and biocides exist, selective pressure to acquire resistance genes to one may select for resistance genes to the other. Therefore, the misuse of biocides may contribute to a potential risk for the development of antibiotic resistance. As a consequence, effective treatment of infections with commonly used antibiotics may fail, increasing the risk of invasive illness or death [18,31,32].

Over the past several decades, there has been an increased emergence of MDR bacteria, defined as non-susceptibility to at least one agent in three or more antimicrobial categories [33]. This includes pathogens such as *Salmonella* and other bacteria causing a serious threat to public health worldwide [34,35,36,37,38,39]. The MDR bacteria have the possibility for transfer to humans via contaminated foods, potentially resulting in untreatable infections. The CDC estimated that approximately 100,000 persons are sickened and 40 deaths occur annually as a result of infection with antibiotic-resistant NTS in the United States [40]. There is no clear evidence that emergence of antibiotic resistant bacteria are due to the use of biocides. However, laboratory based studies suggest that there are associations between antibiotic and biocide resistance [24,25,41,42,43,44]. This may be because biocide-resistant organisms in laboratories are developed using higher than recommended concentrations of biocides so these organisms may not be representative of *in situ* conditions [15,18,45]. Currently there is not enough field survey data to provide clear conclusions that the use of biocides is related to emergence of antibiotic resistant bacteria. In addition, some studies have suggested that bacteria acquired antibiotic resistance first and then became resistant to biocides [46,47,48]. Other studies showed that there was no cross-resistance between the susceptibility of antibiotics and disinfectants among the *Salmonella* isolated from turkeys in commercial processing plants or from feedlot water-sprinkled cattle.[49,50]. Higher MICs in antibiotic resistant bacteria for biocides has been reported whereas biocide-tolerant bacteria have also been found to be more susceptible towards antibiotics [47]. Other studies reported that clinical isolates were more antibiotic-resistant than isolates from industrial environments based on the Total Susceptibility Index and found correlations between antibiotic and biocide resistance in clinical strains, but not in industrial isolates [46,51]. These conflicting results have caused confusion on this subject.

As there is no standard method available for biocide susceptibility testing, the primary objective of this study was to develop a biocide panel to determine the susceptibility of *Salmonella* isolates to 17 commonly used commercial poultry processing and/or household biocides in a single assay. A secondary objective was to determine if a correlation between biocide and antimicrobial resistance was observed. The biocide panel contained two-fold serial dilutions of quaternary ammonium compounds (QACs) (DC, BKC, CPC, HB, HC); organic acids (AA, LA, CA, PXA, ASC); biguanides (CHX); halogen based compounds (SHB, DBH); alkaline compounds (SM); phosphate based compounds (TSP); and arsenical compounds (ARI, ARA). The panel was used to determine the MICs of antimicrobial susceptible negative control strains, quaternary ammonium resistant positive control strains, and 88 MDR *Salmonella* isolates.

## Materials and methods

### Preparation of biocide solutions

Dodecyltrimethylammonium chloride, benzalkonium chloride, cetylpyridinium chloride, hexadecyltrimethylammonium bromide, hexadecyltrimethylammonium chloride, lactic acid, citric acid, peroxyacetic acid, sodium chlorite, 1,3 dibromo, 5,5 dimethylhydantoin, chlorhexidine, sodium metasilicate, trisodium phosphate, and sodium arsenite, were purchased from Sigma Aldrich Laboratories, Inc. (St. Louis, MO, USA). Acetic acid was obtained from Spectrum (Spectrum Chemical & Laboratories Products, Gardena, CA, USA) and sodium arsenate purchased from MP Biomedicals (Solon, OH, USA). Sodium hypochlorite was Clorox brand regular bleach purchased from a retail grocery store. All stock solutions were prepared in autoclaved de-ionized water. Chemicals which were partially soluble in water (CPC, DC, BKC, TSP, HB, and HC) were incubated in hot water for 10 min (~95°C) and vortexed. CHX was dissolved in mixture of isopropanol (6ml) and ethanol (2ml), incubated in hot water for 1h and made the volume 50 ml with water. SM was also incubated in hot water for 1 h and vortexed frequently. DBH was dissolved in 0.5 ml ethyl alcohol and left at room temperature for 15 minutes until it turned orange and then water was added to achieve the final desired concentration. ASC solution was prepared by mixing sodium chlorite (SC) solution with acetic acid to a final pH 3 to 3.5. All stock solutions except CHX, DBH, and SHB were filter sterilized using a 0.2 μm pore size, 25mm sterile syringe filter (Nalgene, Rochester, NY, USA) and were diluted with equal volume of 2x Mueller Hinton broth (MHB) (Thermo Fisher Scientific, Pittsburg, PA, USA). The 2- fold serial dilutions of each chemical was prepared in 1x MHB.

### Preparation of biocide panel

**Fig 1:**
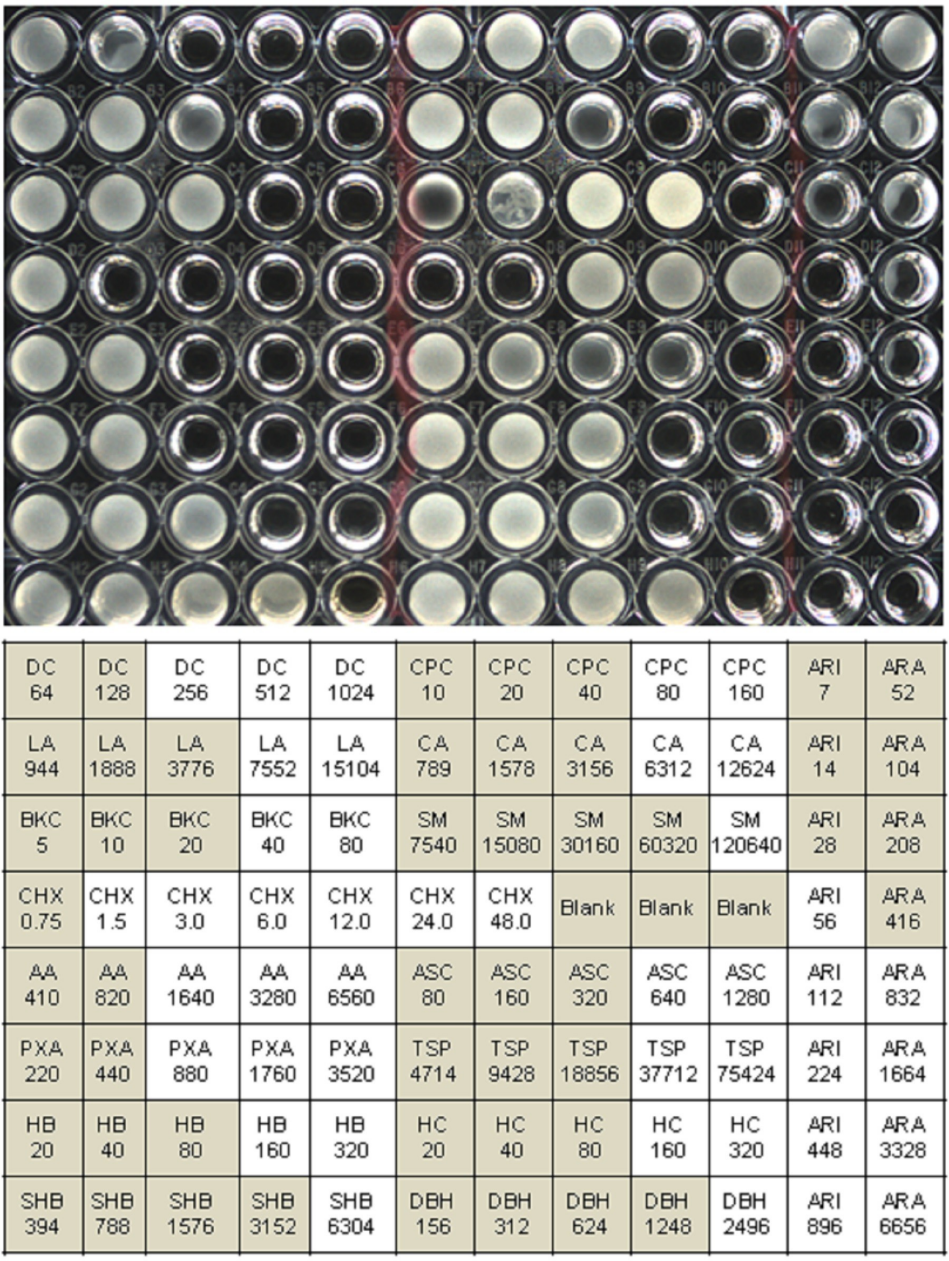
Example of 96 well biocide panel: Susceptibility assay of *S*. Heidelberg using 17 biocides panel. Panel A is an image of biocide panel inoculated with *S*. Heidelberg after 18–24 hrs incubation. Panel B is showing the distribution of 2-fold serial dilutions of 17 biocides in 96-well plate and interpretation of results. Gray region (turbid well) is indicating growth and white region (clear well) is indicating no growth.

Throughout the design, development, and use of this assay, CLSI methods were followed as outlined in the CLSI methods manual [52]. The Minimum Inhibitory Concentration (MIC) is defined as the lowest concentration tested of the biocide at which no visible bacterial growth was observed. The concentration range of each biocide was selected based on preliminary MIC bioassays using 2-fold serial dilution of a wide range of concentrations of each biocide in a sterile 96-well, clear flat bottom microplate with lid (Product #353072, Falcon, Thermo Fisher Scientific). Five two-fold dilutions were selected for each biocide except ARA, ARI and CHX for which 7–8 two-fold dilutions were used. As the MIC_50_ became apparent from the initial assay results, dilution ranges were adjusted to best bracket the MIC_50_; therefore, some plates had different ranges of dilutions. The 2-fold serial dilutions of each chemical were prepared in 50 ml centrifuge tubes. Dilutions were prepared at twice the required final concentration because addition of equal volume of inoculum in MHB reduced the concentration in half to yield the desired final concentration. From each dilution, 100 μl was dispensed into sterile 96 well plates and more than 100 plates containing all 17 chemicals were prepared (Fig 1). All plates were sealed using adhesive foil (cat No. 60941–074, VWR, Randnor, PA, USA) and frozen at -20°C until used. This type of assay design is analogous to those that determine susceptibility to antibiotics and report an MIC for an unknown isolate to multiple antibiotics using a broth microdilution method (e.g. Sensititer).

### Isolates

Eighty-eight MDR *Salmonella* isolates were obtained from the National Antimicrobial Resistance Monitoring System (NARMS) bacterial culture collection located in the Bacterial Epidemiology and Antimicrobial Resistance Research Unit, Athens, GA. The bacteria were isolated by NARMS partners, and originated from animal sources including cattle, swine, chickens, turkeys, horses, dogs, cats, and retail meat during 1998–2011. *Salmonella* from food animals were isolated from carcass swabs, carcass rinses, or ground meat by inspectors of the Food Safety Inspection Service (FSIS). Isolates from companion animals were from the National Veterinary Services Laboratories (NVSL), Ames, IA. One isolate of serovar Heidelberg was from a human infection associated with the 2011 multistate outbreak linked to ground turkey in the United States [53,54]. The identity of animal and human isolates were made anonymous before submission to the culture collection. Isolates were serotyped with Pulsed-Field Gel Electrophoresis (PFGE) and SMART-PCR (Salmonella Multiplex Assay for Rapid Typing) and the Kauffman and White method [55,56]. Antimicrobial susceptibility was previously determined using custom-made broth microdilution plate CMV3AGNF for the theSensititre System following the manufacturer’s instructions (Thermo Fisher, USA). Results were interpreted following Clinical and Laboratory Standards Institute (CLSI) guidelines [57].

Three mutant strains with reduced susceptibility (SRS) to DC and the reference (parental) strain they were derived from L1, L2, L3, and ATCC 4931 were provided by Dr. Diane Herson, U. Delaware and were used as positive controls with the biocide panel. These SRS strains were previously developed from the reference strain *Salmonella enterica* subsp. *enterica* serovar Enteritidis ATCC 4931 by sub-culturing the reference strain in increasing concentrations of DC [58,59,60]. As a MDR positive control, *S*. *enterica* serovar Typhi CT18, MDR including ampicillin [61] and a MDR negative control *S*. *enterica* serovar Typhimurium LT2, ampicillin susceptible [62] was obtained from the American Type Culture Collection (ATCC, Manassas, VA) [63] were also tested for biocide susceptibility.

### Preparation of isolates

The isolates were streaked from -80°C frozen glycerol stocks onto Mueller Hinton agar plates and incubated at 37°C for 18–24 h. From this plate, 3–4 colonies were selected and suspended in Mueller Hinton broth (MHB). Turbidity of inoculums was adjusted to 10^8^ cells ml^-1^ using 0.5 McFarland standards and diluted to a final concentration of inoculum 10^5^ cells ml^-1^.

### Minimum inhibitory concentrations (MIC)

Frozen plates were thawed at room temperature and inoculated with 100 μl of 10^5^ cells ml^-1^. The plates were then covered with a sterile lid and incubated for 18–24 h at 37°C. Growth of bacteria was observed visually; turbid wells indicated growth and clear wells were recorded as no growth (Fig 1). The MIC was recorded as the lowest concentration of biocide at which no visible bacterial growth was observed. The MIC_50_ (median MIC) and MIC_90_ represented the MIC at which ≥50% and ≥90% of all of the isolates were susceptible, respectively. The median MIC_50_ for all isolates was defined as the breakpoint for each chemical, the median used as the MIC is a interval-censored variable and not a continuous variable. A difference of one 2-fold dilution factor MIC (+/-) from the breakpoint was considered allowable variation, therefore, isolates with ≥ two 2-fold dilutions MICs higher than the breakpoint was considered to have reduced susceptibility (“resistance”) to that compound; isolates with MICs ≤ two 2-fold dilutions lower than the breakpoint were considered hyper susceptible.Each batch of plates was tested with negative control strains LT2, CT18, and ATCC4931, and also SRS positive control strains L1, L2, and L3 which are resistant to DC. Any batch of plates that did not give the expected results +/- one 2-fold dilution with these control strains were discarded and all assays were repeated with new plates. This step was done to detect pipette error during dilutions and/or replicate plating and to detect the decay of any of the compounds during storage. Any batch of plates with greater than one 2-fold dilution MIC change for any chemical for the control strains were discarded, after the developmental stage, no plate batches failed this test. Each experimental isolate was tested two to four times with different plate batches, on different days to ensure reproducibility. Data was accepted only if the two assays determined that the MIC differed by less than one 2-fold dilution.

## Results and discussion

Biocides are essential for controlling the spread of pathogens and are beneficial for public health. The effectiveness of biocides depends on several factors such as concentration, status of bacteria (biofilm or planktonic) and presence of interfering materials such as organic matter. Bacteria in biofilms are more resistant to disinfectants than their planktonic counterparts because bacteria in biofilm are less permeable than those of free bacteria [64,65,66]. The most important factor in controlling pathogens is the concentration of biocide used which should be determined cautiously alongside other physical and chemical factors such as organic load, humidity, pH, and temperature [18,67,68]. The unnecessary use of high concentration of biocides could be an environmental hazard whereas sub-inhibitory concentrations of biocides may contribute to the development of resistant bacteria posing a public health hazard. Therefore, it is important to determine the minimum concentration of biocide needed to stop the growth of bacteria and this testing should be performed more frequently. The effective use of biocides also depends on selection of the most suitable biocide for a particular environment because different microorganisms vary in their response to different biocides due to their intrinsic properties and impermeability [69,70,71]. For example, Gram-positive bacteria are more susceptible to biocides than Gram-negative bacteria because of the structure of the outer membrane of Gram-negative bacteria which acts as a permeability barrier [72]. It is important to understand the mechanism of action of biocides to improve the antimicrobial formulation and to prevent the emergence of resistance in bacteria [73]

Standard methods are available for antibiotic susceptibility testing of bacteria. One of the most widely used method is broth microdilution which allows approximately 12–17 antibiotics to be tested in a range of 8 two-fold dilutions in a single 96 well plate [74,75]. However, there is no standard method available for biocide susceptibility testing. In the present study, we adopted the broth microdilution method for testing susceptibility to biocides and designed a 96 well plate to accommodate 17 biocides containing five two-fold dilutions of each biocide except ARI, ARA, and CHX which used seven to eight two-fold dilutions. The concentration range of each biocide was selected based on the results of preliminary susceptibility testing of a wide range of concentrations of each biocide starting at the limit set by Food and Drug Administration (FDA) for use in processing, and diluting from there (data not shown). The biocides selected for susceptibility testing belong to diverse groups of chemicals including quaternary ammoniums, organic acids, biguanides, halogens, alkaline, phosphate, and arsenical-based compounds. These compounds were selected for the biocide susceptibility testing panel because they are widely used antimicrobial interventions as disinfectants in household, poultry, and healthcare facilities as well as preservatives in cosmetics and food industries. The U.S. Food and Drug Administration (FDA) has approved most of these biocides in poultry processing plants as GRAS (generally recognized as safe) substances [76]. The MIC of 95 *Salmonella* isolates including 88 MDR isolates from the NARMS collection was determined using the designed biocides panel as depicted in Fig 1. The biocides’ MICs for the isolates were determined more than once to find the best concentration range of each biocide suitable for analyzing different isolates. However, the results shown here are from one replicate of MICs determination using final designed 96 well panels of 17 biocides. The precision of this method is plus or minus one two-fold concentration which can likely be improved by automating all processes robotically. Currently no Clinical and Laboratory Standards Institute (CLSI) guidelines have been defined for biocide testing and also no criteria for categorization of bacteria as susceptible or resistant to a particular biocide. Since there is no reference breakpoint MICs available for biocides, we recorded the MIC_50_ as the breakpoint MIC. Bacteria were categorized as susceptible if the MIC was equal or lower than the MIC_50_ and defined as resistant if the MIC was 4-fold (two 2-fold dilutions) greater than the MIC_50_. All isolates from the NARMS collection used in this study met the criteria to be defined as MDR as they were resistant to a minimum of 2 and a maximum of 13 antibiotics tested.

### Quaternary ammonium compounds, QACs

**Fig 2:**
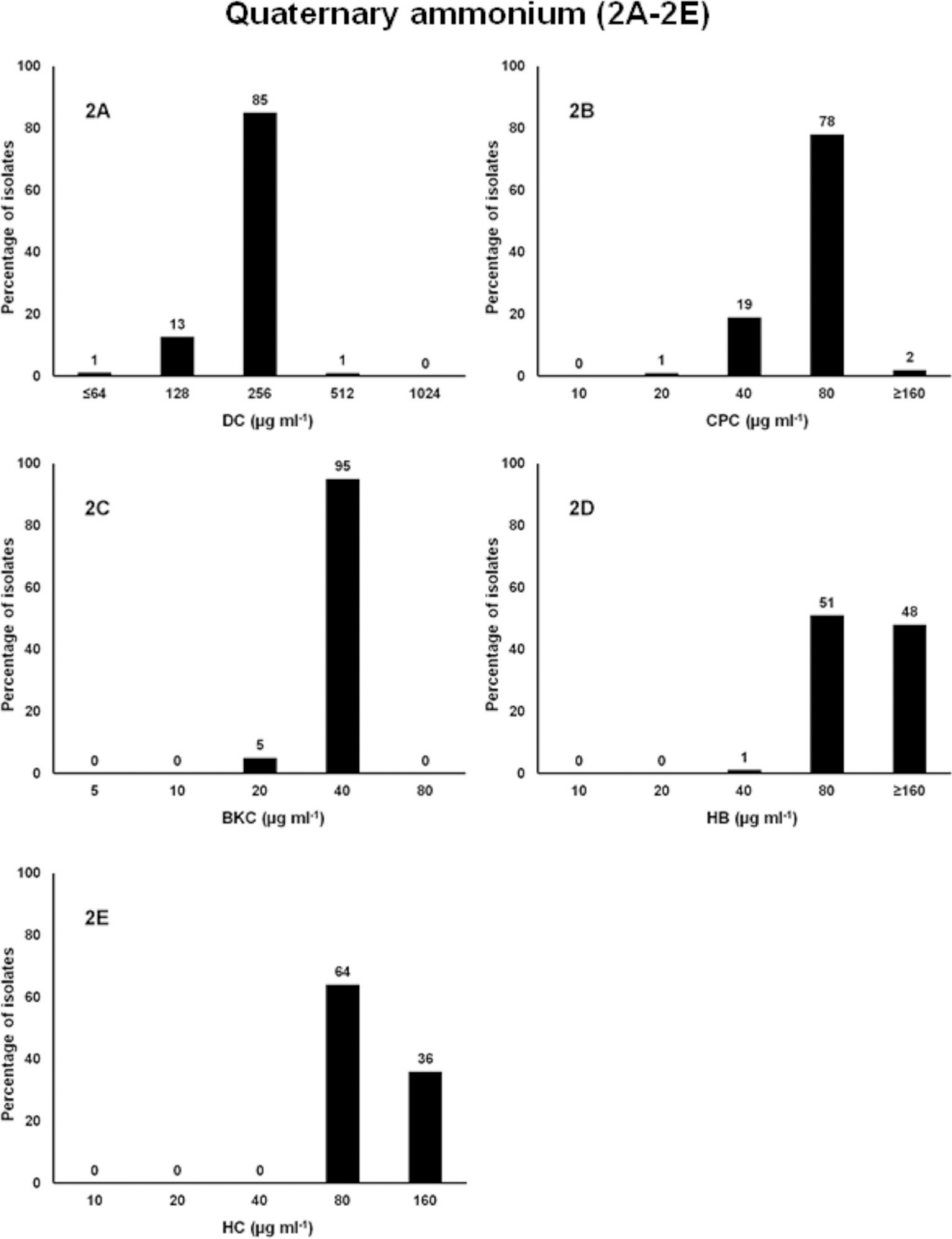
Frequencies of the MICs of the 88 MDR *Salmonella* isolates to quaternary ammonium compounds (QAC). X-axis is the 2-fold increase of biocide concentration; y-axis is percentage of isolates susceptible at each concentration of the QAC tested. 2A. Dodecyltrimethylammonium chloride, DC; 2B. Cetylpyridinium chloride, CPC; 2C. Benzalkonium chloride, BKC; 2D. Hexadecyltrimethylammonium bromide, HB; and 2E. Hexadecyltrimethylammonium chloride, HC.

These compounds are cationic surfactants which primarily target the cytoplasmic membrane of bacteria. They possess antimicrobial activity against a broad range of microorganisms and are widely used in clinical facilities, households, and also in numerous industrial and pharmaceutical products as preserving agents [77,78]. The susceptibility of five QACs (DC, BKC, CPC, HB, and HC) were tested against 88 MDR *Salmonella* isolates using concentrations ranging from 5 to 1024 μg ml^-1^ (0.0005–0.10%). Results showed that 99% to 100% of the isolates were susceptible to all 5 QACs at either the MIC_50_ or 2-fold higher concentration than the MIC_50_ (Fig 2A–2E, Table 1). The MIC_50_ for DC was 256 μg ml^-1^ at which growth of all isolates except one was inhibited (Fig 2A). One isolate from swine, resistant to 11 antibiotics, was susceptible at 2-fold higher concentration (512 μg ml^-1^) than the MIC_50._ The MIC of one SRS with reduced susceptibility to DC was 2-fold higher than the MIC_50_ and two other SRS were not inhibited at the maximum concentration tested (512 μg ml^-1^) and could be considered resistant. The MIC of the reference (parental) strain *Salmonella* enterica subsp. *enterica* serovar Enteritidis ATCC 4931 was 2-fold lower than the MIC_50_. The MIC of SRS and the reference strain is in agreement with a previous report [58]. DC affects the bacterial membrane causing leakage of cell contents resulting in cell death [59]. The MIC_50_ for CPC was 80 μg ml^-1^ at which 98% of the isolates were susceptible (Fig 2B). Growth of an *S*. Heidelberg isolated from turkey carcass, resistant to 5 antibiotics was not inhibited at the highest CPC concentration (160 μg ml^-1^) tested and was categorized as resistant to CPC. The maximum concentration of CPC approved by the FDA to treat the surface of raw poultry carcasses with a solution contains a maximum of 0.8% (8000 μg ml^-1^) CPC, followed by a potable water rinse or immersion in chiller water [79]. The breakpoint MICs found for MDR *Salmonella* in this study was 100 times lower than that allowed by the FDA. The MIC_50_ for BKC was 40 μg ml^-1^ at which 100% of the *Salmonella* was susceptible (Fig 2C). The MIC_50_ for both HB and HC was 80 μg ml^-1^ and 99% of isolates for HB and 100% of isolates for HC were susceptible at 2-fold higher concentration (Fig 2D and 2E) than the MIC_50._ An *S*. Agona isolated from swine in 2005, resistant to 11 antibiotics was also resistant to HB. This isolate was also resistant to CHX and was the only isolate with 2-fold higher MIC than the MIC_50_ for DC. It is possible that the repeated exposure of bacteria to QAC biocides could reduce susceptibility to other biocides or to antibiotics which may be due to temporary phenotypic adaptations or the selection of stable genetic mutations [44].

**Table 1:** Minimum inhibitory concentration (MIC) of 17 disinfecting chemicals / biocides for 88 MDR *Salmonella* isolates.

Biocides^a^	Range tested (μg ml^-1^)	MIC _50_ (μg ml^-1^)	MIC _90_ (μg ml^-1^)	% Isolates susceptible at MIC _90_
**DC**	32–512 or 64–1024	256	256	99
**CPC**	10–160	80	80	98
**BKC**	5–80	40	40	100
**HB**	10–160 or 20–320	80	160	99
**HC**	10–160 or 20–320	80	160	100
**AA**	410–6560	1640	3280	100
**LA**	944–15104	5664	7552	100
**CA**	789–12624	3156	12624	100
**PXA**	220–3520	880	1760	100
**ASC**	80–1280	320	640	92
**CHX**	0.75–48.0	3.0	12.0	100
**DBH**^**b**^	156–2496	1248	2496	100
**SHB**^**c**^	394–6304	3152	6304	100
**SM**	7540–120640	60320	120640	100
**TSP**	4714–75424	37712	37712	100
**ARA**	52–6656	832	3328	88
**ARI**	7–896	56	112	96

^a^ DC- Dodecyltrimethylammonium chloride; CPC- Cetylpyridinium chloride; BKC- Benzalkonium chloride; HB- Hexadecyltrimethylammonium bromide; HC- Hexadecyltrimethylammonium chloride; AA- Acetic acid; LA- Lactic acid; CA- Citric acid; PXA- Peroxyacetic acid; ASC- Acidified sodium chlorite; CHX- Chlorhexidine; DBH- 1,3 dibromo, 5,5 dimethylhydantoin; SHB- Sodium hypochlorite; SM- Sodium metasilicate; TSP- Trisodium phosphate; ARI- Sodium arsenite; ARA- Sodium arsenate. The MIC_50_ and MIC_90_ were defined as the biocide concentrations that inhibited growth of 50 and 90% of the isolates respectively.

^b^ DBH data shown here is for 65 isolates

^c^ Actual hypochlorite concentration in household bleach. Clorox brand household bleach is a solution of 5.25% or 52,500 ppm of sodium hypochlorite.

QAC resistance genes were found among *Escherichia coli* isolated from retail meat and associated with MDR phenotypes suggesting that QACs in the food industry may not be as effective as expected and bacteria could develop resistance to other antimicrobials [80]. The recommended concentration of QACs when used as disinfectants is below 1,000 μg ml^- 1^ [81] and is a concentration at which most of our tested strains would not grow.

### Organic acids

**Fig 3:**
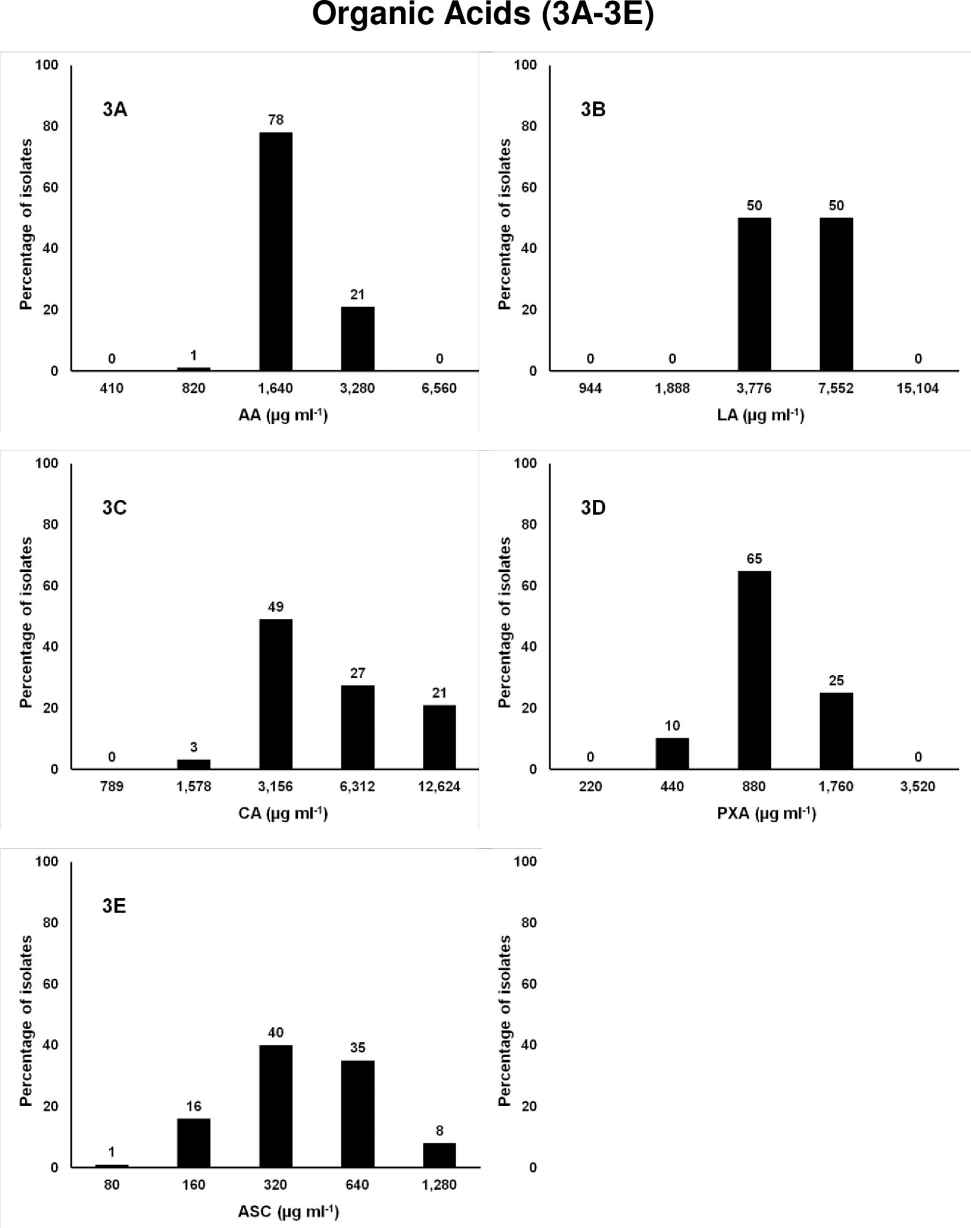
Frequencies of the MICs of 88 MDR *Salmonella* isolates to organic acids. X-axis is the 2-fold increase of biocide concentration; y-axis is percentage of isolates susceptible at each concentration of organic acids. 3A. Acetic acid, AA; 3B. Lactic acid, LA; 3C. CA, Citric acid; 3D. Peroxyacetic acid, PXA; and 3E. Acidified sodium chlorite, ASC.

These chemicals work by lowering the intracellular pH causing damage to outer or cytoplasmic membrane resulting in bacterial death [82,83]. They are widely used in the poultry industry as additives for drinking water or in feed [84,85,86]. The USDA Food Safety and Inspection Service (FSIS) allows 1.5 to 2.5% solutions of most of the organic acids for pre-chilled carcasses washing in commercial plants for both beef and lamb processing [87]. Susceptibility of MDR *Salmonella* isolates was tested against five organic acids LA, CA, AA, PXA, ASC using concentrations ranging from 80 to 15,104 μg ml^-1^, 0.01–1.5% (Fig 3A–3E, Table 1). These organic acids are GRAS at recommended concentrations and are approved by the FDA in food for human consumption [88,89]. They are highly soluble and cannot accumulate in organisms or in the environment [90]. Acetic acid (vinegar in the U.S. sold as 5% acetic acid) is active against most bacteria [77,80]. The MIC_50_ for AA and PXA was 1,640 and 880 μg ml^-1^, respectively, at which 75 to 79% of isolates were susceptible and the remaining isolates were susceptible at a 2-fold higher concentration. The MIC of CA and ASC showed variation between isolates; 52–57% of isolates were susceptible at 3,156 μg ml^-1^ for CA and 320 μg ml^-1^ for ASC which is the median MIC. Another 21% of isolates for CA and 8% of isolates for ASC were resistant with a 4-fold higher MIC than the MIC_50_. The MIC_50_ for LA was 5,664 μg ml^-1^ (average of two MICs), because 50% of bacteria susceptible at 3,776 and other 50% were susceptible at 2-fold higher concentration (7,552 μg ml^-1^).

SC is commonly used in poultry processing water as a spray or dip solution at concentrations between 500 and 1,200 μg ml^-1^ combined with a GRAS acid to achieve a pH ranging from 2.3 to 2.9 [79,91,92]. The concentration and pH of ASC significantly affect its antimicrobial activity [93]. It is commercially supplied as a kit containing citric acid (CA) and SC which are combined to produce active chlorine dioxide. We have used acetic acid instead of citric acid to lower the pH. It has been shown that the type of acid used to lower the pH does not have an effect on its activity [94]. Several laboratory studies have reported reduction in pathogenic bacteria using ASC on carcasses and surfaces of fresh beef [95,96,97].

Peroxiacetic acid (PXA), is a strong oxidizing agent and effective under a wide variety of conditions. It affects the bacterial cell wall permeability and denatures proteins, enzymes, and inhibits other cellular activities. According to the CDC, PXA inactivates both gram-positive and gram-negative bacteria as well as fungi, and yeasts in < 5 minutes at <100 μg ml^-1^. Treatment with PXA showed reduction of pathogenic bacteria on beef carcasses [96,98]. The USDA-FSIS permits the use of PXA at concentrations of 220 μg ml^-1^ on poultry products in extended dwell time chillers and at concentrations of 2,000 μg ml^-1^ short dwell time post-chill dip tanks. Biofilm containing *Campylobacter jejuni* were tested and found to be highly susceptible to PXA treatments [99]. In this study, 75% of *Salmonella* isolates were susceptible at 880 μg ml^-1^ and the remaining 25% were susceptible at 1,760 μg ml^-1^, which is higher than recommended by the CDC. This may be due to the presence of high organic matter in MH broth, as it has been reported that antibacterial activity of PXA can be reduced in the presence of organic matter [94].

### Bigunide

**Fig 4:**
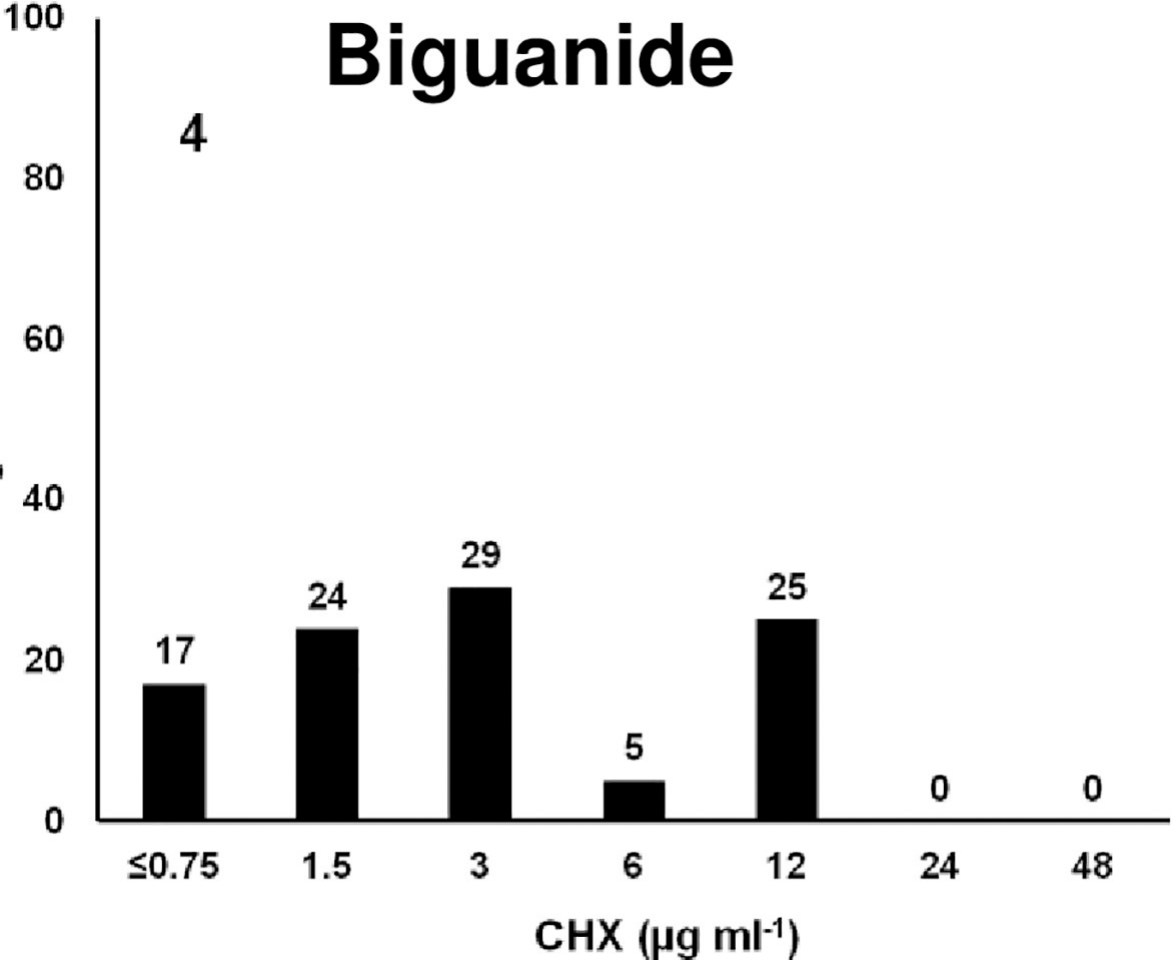
Frequencies of the MICs of 88 MDR *Salmonella* isolates to the biguanide, Chlorhexidine, CHX. X-axis is the 2-fold increase of biocide concentration; y-axis is percentage of isolates susceptible at each concentration of CHX.

The bigunide biocide used was CHX which is widely used as a disinfectant, an antiseptic and a preservative in both medical and agricultural environments, and in domestic cleaning products [100]. It is also used in food industries, but is most commonly used in hand sanitizers, usually at a concentration of 2 to 4% [101]. It is effective against a wide range of both gram-positive and gram-negative bacteria causing damage to the cell membrane [101]. Despite the advantages of CHX, its activity is pH dependent and is greatly reduced in the presence of organic matter. The MIC for these isolates ranged widely, from <0.75 ug ml^-1^ to 12 μg ml^-1^. Results showed that 70% of isolates were susceptible at MIC_50_ (3.0 μg ml^-1^) and 25% were resistant with 4 fold higher MIC compared to the MIC_50_(Fig 4). The MIC of CHX for 70% isolates was in similar range as found previously which showed that 85% of Salmonella isolates from feedlot water-sprinkled cattle were susceptible at 4 μg ml^-1^ [49].

Six out of seven control strains were resistant to CHX, with the reference strain to the DC resistant strains being the only sensitive control at 3.0 μg ml^-1^. Two control strains which were resistant to DC showed highest MIC for CHX (24 μg ml^-1^) and the reference strain was susceptible to 2-fold below the MIC_50_ for both DC and CHX. DC and CHX both target the cell membrane, so it is possible that strains with resistance to DC would also be resistant to CHX. More interestingly, 8/9 *S*. Newport isolates were CHX resistant, and most of these were cattle associated isolates. These CHX resistant *S*. Newport were also resistant to ARA, as discussed below. Six out of nine *S*. Typhimurium were CHX resistant, and three of these were also ARA resistant, however, the Typhimurium were isolated from a variety of sources. All four *S*. Agona isolates were resistant to CHX. Three of the *S*. Agona were from cattle and one was from swine. Finally, four out of seven *S*. Dublin were resistant to CHX, with three isolated from cattle and one from chicken.

### Halogen-based compounds

**Fig 5:**
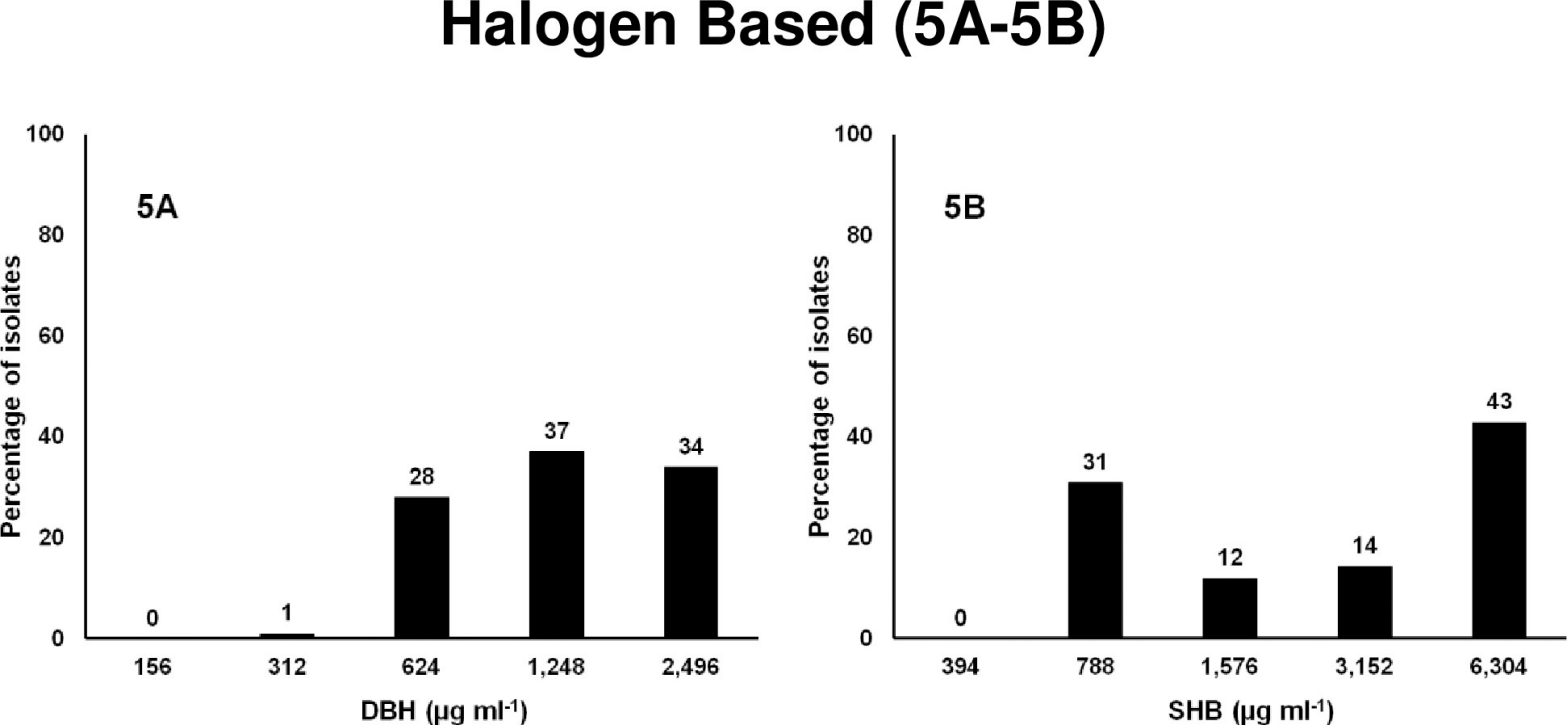
Frequencies of the MICs of 88 MDR *Salmonella* isolates to halogen based compounds. X-axis is the 2-fold increase of biocide concentration; y-axis is percentage of isolates susceptible at each concentration. 5A. 1,3 dibromo, 5,5 dimethylhydantoin, DBH and 5B. Sodium hypochlorite, SHB.

In this study, susceptibility of *Salmonella* was also tested against chlorine and bromine based biocides. Household bleach is an aqueous solution of 5.25%–6.15% sodium hypochlorite containing 52,500 to 61,500 μg ml^-1^ available chlorine. We used Clorox brand bleach which was 5.25% sodium hypochlorite. It is a powerful oxidant and has a broad spectrum of antimicrobial activity [102,103] and induces lysis in gram-negative bacteria by affecting the cell wall [73]. The MIC of SHB in this study showed variation between isolates. The MIC_50_ was 3,152 μg ml^-1^ (6%) at which 57% of isolates were susceptible. The other 43% were susceptible at a 2-fold high concentration (12%) which is slightly higher than the recommended concentration 10% (v/v) solution (Fig 5B). We also observed that the MIC of isolates was below 6% when a fresh bottle of bleach was opened. The free available chlorine levels of hypochlorite solutions reduced to 40% to 50% of the original concentration over a period of one month at room temperature in both opened and closed polyethylene containers (https://ehs.colorado.edu/resources/disinfectants-and-sterilization-methods/).

The bromine disinfectant biocide (DBH) hydrolyzes in water to form the active antimicrobial compound, hypobromous acid (HOBr), which works within a wider range of pH and is also more stable than SHB in the presence of organic matter. It also does not cause corrosion to plant equipment or concrete floors. DBH is not GRAS by FDA, but is used in post-slaughter processing [104]. The aqueous solution is allowed up to 100 ppm on poultry carcasses, 300 ppm on beef and 500 ppm on swine. DBH can also be used in water to make ice for poultry and beef processing [105]. It reduces *Salmonella* contamination in hides when treated with 220–500 ppm of HOBr. In this experiment MIC of 65 isolates were determined using 156 to 2496 μg ml^-1^ and MIC of remaining 23 isolates were determined using 228 to 3648 μg ml^-1^.Results showed that the MIC_50_ of 65 isolates was 1248 μg ml^-1^ at which 66% of isolates were susceptible while the remaining 34% were susceptible at a 2-fold higher concentration (Fig 5A). The MIC_50_ of other 23 isolates was 3648 at which all 23 isolates were susceptible (data not shown). The MIC of DBH is 2 to 5 fold higher in this study than the maximum concentration recommended by FDA. It may also be possible that this solution is not stable in water at room temperature and loses its active bromine.

### Alkaline compounds

**Fig 6:**
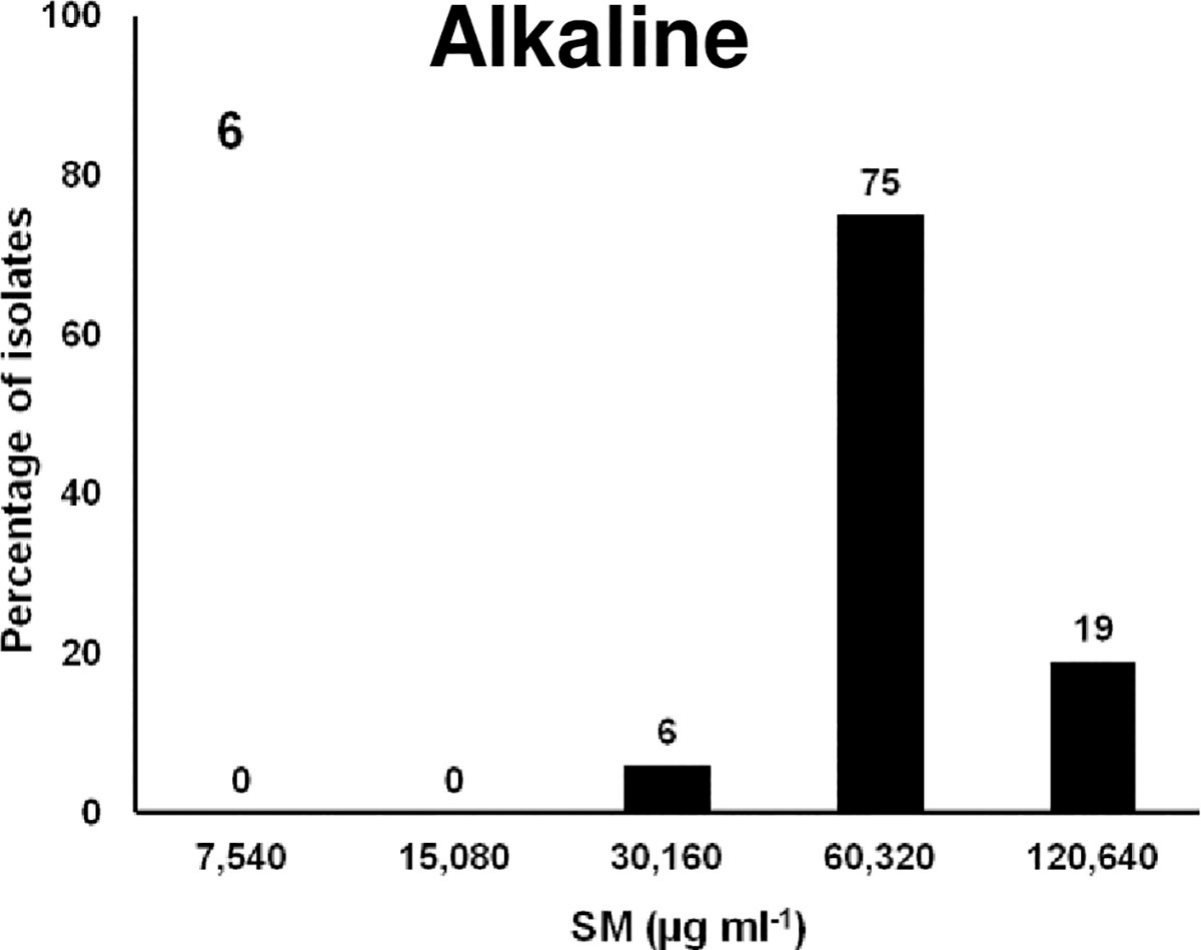
Frequencies of the MICs of 88 MDR *Salmonella* isolates to alkaline compound, Sodium metasilicate, SM. X-axis is the 2-fold increase of biocide concentration; y-axis is percentage of isolates susceptible at each concentration of SM.

Sodium metasilicate is considered GRAS and is permitted to be added directly to food for human consumption [106]. It was approved by USDA-FSIS in 2012 as an alkaline antimicrobial component of marinades for poultry [107]. It is ineffective after neutralization. SM acts on the cytoplasmic membrane and causes lysis of the cells and leakage of intracellular contents. Susceptibility of *Salmonella* isolates was tested against SM using a 2- fold serial dilution ranging from 7,540 to120,640 μg ml^-1^ (1 to 12% SM). Results showed that the MIC_50_ was 60320 μg ml^-1^ (6%) at which 81% of isolates were susceptible; the remaining 19% were susceptible at a 2-fold higher concentration (Fig 6). The MIC of the isolates in this study was higher (6 to 12%) than the USDA-FSIS maximum approved level of 2% for poultry. The disadvantages of the chemical include discoloration of the fillets and a suitable pH for microbial growth [108,109].

### Phosphate based compound

**Fig 7:**
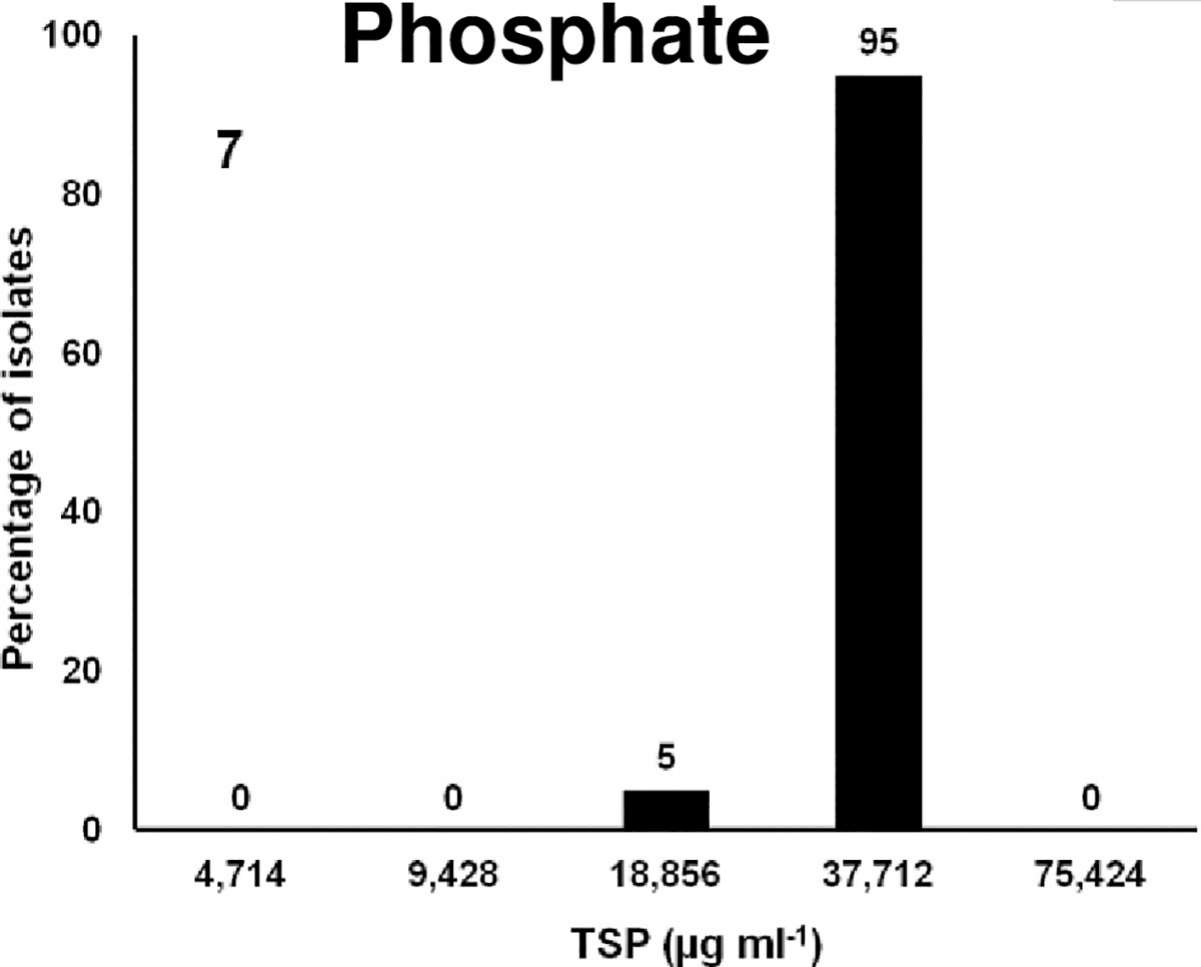
Frequencies of the MICs of 88 MDR *Salmonella* isolates to phosphate compound Trisodium phosphate, TSP. X-axis is the 2-fold increase of biocide concentration; y-axis is percentage of isolates susceptible at each concentration of TSP.

Trisodium phosphate is recommended as GRAS by the USDA-FSIS. The allowed concentration of TSP is 8 to 12% (80,000–120,000 μg/ml) to decontaminate poultry carcasses either by spraying or dipping in the solution [110,111]. It is more effective on gram-negative bacteria than against gram-positive. It removes bacteria that are not firmly attached to the skin surface and also removes some surface fat which facilitates the removal of bacteria by the washing process [112]. Several studies have shown that TSP treatment of poultry carcasses and meat effectively reduced various pathogenic bacteria [113,114]. In this study, the MIC_50_ was 37,712 μg ml^-1^ at which 100% of isolates were susceptible (Fig 7 and Table 1). The MIC of *Salmonella* isolates was 50% lower than the allowed concentration.

### Arsenical compounds

**Fig 8:**
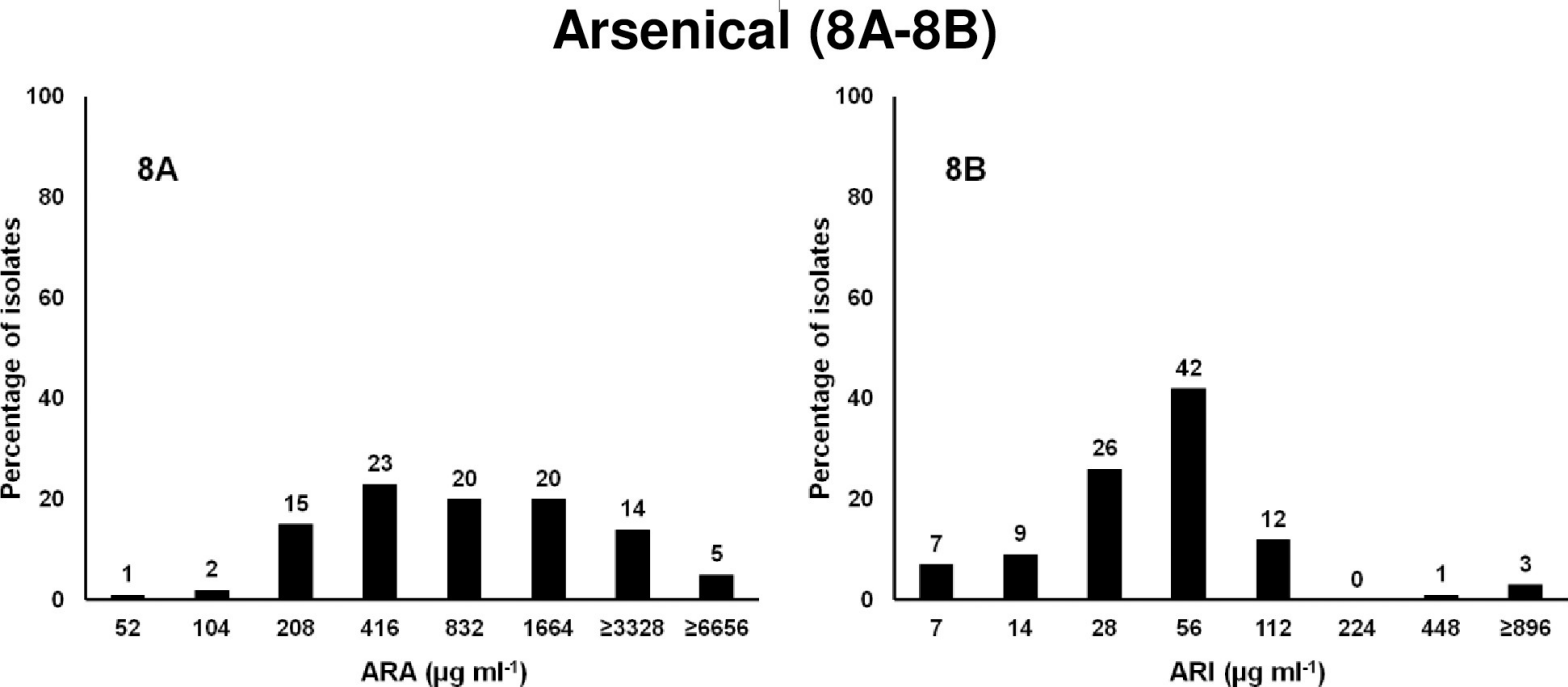
Frequencies of the MICs of 88 MDR *Salmonella* isolates to arsenical compounds. X-axis is the 2-fold increase of biocide concentration; y-axis is percentage of isolates susceptible at each concentration. 8A. ARA, arsenate; and 8B. ARI, aresenite.

Susceptibility of *Salmonella* isolates against two arsenical compounds, ARI and ARA, was tested. Results from our preliminary experiments showed higher variability in MICs of *Salmonella* isolates for both compounds. Eight dilutions were tested instead of 5 ranging in concentration from 52 to 6,656 μg ml^-1^ for ARA (Fig 8A) and 7 to 896 μg ml^-1^ for ARI (Fig 8B). The MIC_50_ was 56 μg ml^-1^ for ARI and 832 μg ml^-1^ for ARA at which 84% (ARI) and 61% (ARA) of isolates were susceptible. The MICs of 4% of isolates for ARI and 19% of isolates for ARA was 4 to >6 fold higher than the MIC_50_ and were categorized as resistant. The MIC of resistant isolates was 6 to 20-fold lower than previously reported for two arsenic resistant bacteria isolated from agricultural soil with an MIC of 400 mM (83,200 μg ml^-1^) for arsenate and 40 mM (5,200 μg ml^-1^) for arsenite [115]. Three of our control isolates were ARA resistant including *S*. Typhi CT18, *S*. Typhimurium LT2, and *S*. Typhimurium 14028. As stated above eight out of nine *S*. Newport isolates were resistant to both ARA and CHX, as well as three out of the five CHX resistant *S*. Typhimurium isolates were ARA resistant. As the targets of ARA and CHX are energy metabolism and membranes respectively, no clear method of cross resistance would be obvious, other than perhaps membrane permiability/transport of the compounds. Arsenical biocides are commonly used as herbicides, insecticides, fungicides, plant growth regulators, and animal feed additives. Both arsenite and arsenate are toxic but arsenite As III is 60 times more toxic than arsenate As V [116]. Only four of these isolates were resistant to ARI and all were resistant to ARA. Arsenic-based animal drugs, such as roxarsone, have been widely used in poultry feed for more than 60 years to promote faster weight gain with less food and also to produce healthy colored meat [117,118,119,120,121]. It was estimated that 9 × 10^5^ kg of roxarsone was excreted in poultry litter each year which degrades into more toxic water soluble inorganic arsenic [122,123,124]. In 2011, an FDA study found higher levels of inorganic arsenic residues in chicken livers which were fed roxarsone-supplemented feeds [120]. In response to this study, the manufacturer of roxarsone in the United states voluntary suspended its supplies [120]. At the end of 2015, the FDA completely banned the use of arsenic-based drugs in food-producing animal feed [120,121]. It was reported that *Campylobacter* spp from conventional poultry showed significantly higher MICs for roxarsone than from antimicrobial-free poultry suggesting bacteria which are exposed to arsenic feed develop resistance towards the arsenic [125] However, MICs of arsenate and arsenite were the same for both locations. This could be because arsenic is one of the most abundant toxins present in the natural environment and many bacteria have resistance pathways to detoxify arsenic [126]. In one system the substrate for the ArsB transport protein and ArsC activity is closely coupled with its efflux from the cells so that intracellular arsenite does not accumulate [127].

## Conclusions

Our custom designed biocide panel provides a rapid method to determine the MICs of 17 biocides for ~50 isolates in less than 48 hours. This biocide plate can be produced in high volume using an automatic dispenser and can be frozen for future use. The biocide panel would also be useful in evaluating incremental changes in resistance to biocides over time as bacteria that were susceptible may develop resistance. The routine monitoring of MICs will be useful in selecting the most effective and economical antimicrobial agents for decontamination of a particular environment. The panel was used to test the susceptibility of 88 MDR *Salmonella* isolates from animal sources obtained from the animal arm of NARMS. All *Salmonella* isolates in this study were resistant to a minimum of 2 and a maximum 13 antibiotics and no clear correlation of biocide resistance to antibiotic resistance was detected. All biocides were effective in killing bacteria and 98 to 100% of isolates were susceptible at either the MIC_50_ or 2-fold higher concentration than the MIC_50_ except ASC, CA, ARA, ARI, and CHX. A positive correlation was apparent between resistance to ARA and CHX, as well as a correlation between ARA and ARI. The associations between ARA and CHX also seemed to be serovar specific with *S*. Newport, *S*. Agona, and *S*. Dublin demonstrating the most co-resistance to ARA and CHX. The *S*. Typhimurium controls and a few other Typhimurium isolates also showed some ARA and CHX co-resistance. The ARI and ARA showed some correlation with each other but not with other biocides, and there may be correlations between other biocides such as ASC, ARA, and CHX.

### Future work

No breakpoint MICs are defined for biocides to distinguish between resistant or susceptible bacteria. Our study focused on mostly MDR *Salmonella*, thus a randomly selected group of wild type isolates from the NARMS collection would be required to determine true wild type MICs. In addition, more testing of *Salmonella* known to be resistant to biocides would help define “resistant” MICs. Therefore, a future goal is to determine the MICs of a large population of *Salmonella* using the biocide panel to establish breakpoints. More studies of antibiotic resistant and MDR *Salmonella* are also necessary to continue to investigate any linkage between biocide resistance and antibiotic resistance. Additionally, the MICs of bacteria belonging to other important food-safety genera or species need to be established; consequently, the panel and methods can be modified to address different bacteria. Other potential applications for this assay include monitoring development of biocide resistance upon repeated exposure to biocides in processing environments. Monitoring would also detect the development of biocide cross-resistance leading to insights for selective rotation of biocides in processing plants. The correlations between ARA and CHX, and ARA and ARI, as well as other potential associations between different biocides, require further investigation to determine if these resistances are related to each other through mechanism or a genetic linkage. As biocides are important in controlling *Salmonella*, monitoring their efficacy and detecting any development of biocide resistance or links between biocide and antimicrobial resistance would be a very valuable application for this tool.

## Supporting information

S1 Table Minimum inhibitory concentration (MIC) of 17 disinfecting chemicals / biocides in μg ml^-1^ for each isolates with antibiotic resistance (AR), source and year of isolation. (DOCX)

## References

[1] Popoff MY, Bockemühl J, Gheesling LL (2004) Supplement 2002 (no. 46) to the Kauffmann–White scheme. Research in Microbiology 155: 568–570. 10.1016/j.resmic.2004.04.005 15313257

[2] Alcaine SD, Warnick LD, Wiedmann M (2007) Antimicrobial resistance in nontyphoidal Salmonella. Journal of food protection 70: 780–790. 17388077 10.4315/0362-028x-70.3.780

[3] Grimont PA, Weill F-X (2007) Antigenic formulae of the Salmonella serovars WHO collaborating centre for reference and research on Salmonella 9.

[4] CDC (2011) National Enteric Disease Surveillance: Salmonella Surveillance Overview

[5] Andino A, Hanning I (2015) Salmonella enterica: survival, colonization, and virulence differences among serovars. ScientificWorldJournal 520179: 13. 10.1155/2015/520179 PMC4310208 25664339

[6] Newell DG, Koopmans M, Verhoef L, Duizer E, Aidara-Kane A, Sprong H., et al (2010) Food-borne diseases—the challenges of 20 years ago still persist while new ones continue to emerge. International Journal of Food Microbiology 139: S3–S15. 10.1016/j.ijfoodmicro.2010.01.021 20153070 PMC7132498

[7] Altekruse S, Cohen M, Swerdlow D (1997) Emerging foodborne diseases. Emerging infectious diseases 3: 285 10.3201/eid0303.970304 9284372 PMC2627632

[8] Beuchat LR (1996) Pathogenic microorganisms associated with fresh produce. Journal of Food Protection 59: 204–216. 10.4315/0362-028X-59.2.204 31159004

[9] Mead PS, Slutsker L, Dietz V, McCaig LF, Bresee JS, Shapiro C., et al (1999) Food-related illness and death in the United States. Emerging Infectious Diseases 5: 607–625. 10.3201/eid0505.990502 10511517 PMC2627714

[10] Scallan E, Hoekstra RM, Angulo FJ, Tauxe RV, Widdowson MA, Widdowson MA, et al (2011) Foodborne illness acquired in the United States—major pathogens. Emerg Infect Dis 17: 7–15. 21192848 10.3201/eid1701.P11101 PMC3375761

[11] Scharff RL (2012) Economic burden from health losses due to foodborne illness in the United States. J Food Prot 75: 123–131. 10.4315/0362-028X.JFP-11-058 22221364

[12] USDA (2014) Cost Estimates of Foodborne Illnesses, on United States Department of Agriculture, Economic Research Service (ERS). http://ers.usda.gov/data-products/cost-estimates-of-foodborne-illnesses.aspx. Accessed.

[13] Cozad A, Jones RD (2003) Disinfection and the prevention of infectious disease. American Journal of Infection Control 31: 243–254. 12806363 10.1067/mic.2003.49

[14] Carrasco E, Morales-Rueda A, García-Gimeno RM (2012) Cross-contamination and recontamination by Salmonella in foods: A review. Food Research International 45: 545–556.

[15] Møretrø T, Heir E, Nesse LL, Vestby LK, Langsrud S (2012) Control of Salmonella in food related environments by chemical disinfection. Food Research International 45: 532–544.

[16] Hugo W (1995) A brief history of heat, chemical and radiation preservation and disinfection. International biodeterioration & biodegradation 36: 197–217.

[17] Barillo DJ, Marx DE (2014) Silver in medicine: A brief history BC 335 to present. Burns 40: S3–S8. 10.1016/j.burns.2014.09.009 25418435

[18] SCENIHR (2009) SCENIHR (Scientific Committee on Emerging and Newly Identified Health Risks) Assessment of the Antibiotic Resistance Effects of Biocides European Commission, Brussels, Belgium (2009): 1–87.

[19] Sanders FT (2003) The role of the EPA in the regulation of antimicrobial pesticides in the United States. Pesticide Outlook 14: 251–255.

[20] EPA https://www.epa.gov/pesticide-registration/what-are-antimicrobial-pesticides https://www.epa.gov/pesticide-registration/what-are-antimicrobial-pesticides. Environmental Protection Agency.

[21] Capita R, Riesco-Peláez F, Alonso-Hernando A, Alonso-Calleja C (2013) Exposure to sub-lethal concentrations of food-grade biocides influences the ability to form biofilm, the resistance to antimicrobials and the ultrastructure of Escherichia coli ATCC 12806. Applied and environmental microbiology: AEM. 02283–02213. 10.1128/AEM.02283-13 PMC3911067 24317080

[22] Sheridan À, Lenahan M, Duffy G, Fanning S, Burgess C (2012) The potential for biocide tolerance in Escherichia coli and its impact on the response to food processing stresses. Food Control 26: 98–106.

[23] Dynes JJ, Lawrence JR, Korber DR, Swerhone GD, Leppard GG, Hitchcock AP. (2009) Morphological and biochemical changes in Pseudomonas fluorescens biofilms induced by sub-inhibitory exposure to antimicrobial agents. Canadian journal of microbiology 55: 163–178. 10.1139/w08-109 19295649

[24] Karatzas KA, Webber MA, Jorgensen F, Woodward MJ, Piddock LJ, Humphrey TJ. (2007) Prolonged treatment of Salmonella enterica serovar Typhimurium with commercial disinfectants selects for multiple antibiotic resistance, increased efflux and reduced invasiveness. J Antimicrob Chemother 60: 947–955. 10.1093/jac/dkm314 17855722

[25] Molina-González D, Alonso-Calleja C, Alonso-Hernando A, Capita R (2014) Effect of sub-lethal concentrations of biocides on the susceptibility to antibiotics of multi-drug resistant Salmonella enterica strains. Food Control 40: 329–334.

[26] Gantzhorn MR, Pedersen K, Olsen JE, Thomsen LE (2014) Biocide and antibiotic susceptibility of Salmonella isolates obtained before and after cleaning at six Danish pig slaughterhouses. International Journal of Food Microbiology 181: 53–59. 10.1016/j.ijfoodmicro.2014.04.021 24819413

[27] Webber MA, Ricci V, Whitehead R, Patel M, Fookes M, Ivens A, et al (2013) Clinically relevant mutant DNA gyrase alters supercoiling, changes the transcriptome, and confers multidrug resistance. MBio 4: e00273–00213. 10.1128/mBio.00273-13 23882012 PMC3735185

[28] Russell A (2002) Antibiotic and biocide resistance in bacteria: introduction. Journal of applied microbiology 92. 12000607

[29] McCarthy AJ, Lindsay JA (2012) The distribution of plasmids that carry virulence and resistance genes in Staphylococcus aureus is lineage associated. BMC microbiology 12: 104 10.1186/1471-2180-12-104 22691167 PMC3406946

[30] Bennett P (2008) Plasmid encoded antibiotic resistance: acquisition and transfer of antibiotic resistance genes in bacteria. British journal of pharmacology 153: S347–S357. 10.1038/sj.bjp.0707607 18193080 PMC2268074

[31] Helms M, Simonsen J, Mølbak K (2004) Quinolone resistance is associated with increased risk of invasive illness or death during infection with Salmonella serotype Typhimurium. Journal of Infectious Diseases 190: 1652–1654. 10.1086/424570 15478071

[32] Butaye P, Michael GB, Schwarz S, Barrett TJ, Brisabois A, White DG. (2006) The clonal spread of multidrug-resistant non-typhi Salmonella serotypes. Microbes Infect 8: 1891–1897. 10.1016/j.micinf.2005.12.020 16714135

[33] Magiorakos AP, Srinivasan A, Carey R, Carmeli Y, Falagas M, Giske CG, et al (2012) Multidrug‐resistant, extensively drug‐resistant and pandrug‐resistant bacteria: an international expert proposal for interim standard definitions for acquired resistance. Clinical microbiology and infection 18: 268–281. 10.1111/j.1469-0691.2011.03570.x 21793988

[34] Su L-H, Chiu C-H, Chu C, Ou JT (2004) Antimicrobial resistance in nontyphoid Salmonella serotypes: a global challenge. Clinical infectious diseases 39: 546–551. 10.1086/422726 15356819

[35] Angulo FJ, Johnson KR, Tauxe RV, Cohen ML (2000) Origins and consequences of antimicrobial-resistant nontyphoidal Salmonella: implications for the use of fluoroquinolones in food animals. Microbial drug resistance 6: 77–83. 10.1089/mdr.2000.6.77 10868811

[36] Lee LA, Puhr ND, Maloney EK, Bean NH, Tauxe RV (1994) Increase in antimicrobial-resistant Salmonella infections in the United States, 1989–1990. Journal of Infectious Diseases 170: 128–134. 8014487 10.1093/infdis/170.1.128

[37] Karam G, Chastre J, Wilcox MH, Vincent J-L (2016) Antibiotic strategies in the era of multidrug resistance. Critical Care 20: 136 10.1186/s13054-016-1320-7 27329228 PMC4916531

[38] Tanwar J, Das S, Fatima Z, Hameed S (2014) Multidrug resistance: an emerging crisis. Interdisciplinary perspectives on infectious diseases 2014. 10.1155/2014/541340 PMC4124702 25140175

[39] Fair RJ, Tor Y (2014) Antibiotics and bacterial resistance in the 21st century. Perspectives in medicinal chemistry 6: 25 10.4137/PMC.S14459 25232278 PMC4159373

[40] CDC (2013) Antibiotic resistance threats in the United States. http://wwwcdcgov/drugresistance/pdf/ar-threats-2013-508pdf. 25162160

[41] Mc Cay PH, Ocampo-Sosa AA, Fleming GT (2010) Effect of subinhibitory concentrations of benzalkonium chloride on the competitiveness of Pseudomonas aeruginosa grown in continuous culture. Microbiology 156: 30–38. 10.1099/mic.0.029751-0 19815578

[42] Christensen EG, Gram L, Kastbjerg VG (2011) Sublethal triclosan exposure decreases susceptibility to gentamicin and other aminoglycosides in Listeria monocytogenes. Antimicrobial agents and chemotherapy 55: 4064–4071. 10.1128/AAC.00460-11 21746948 PMC3165368

[43] Rakic-Martinez M, Drevets DA, Dutta V, Katic V, Kathariou S (2011) Listeria monocytogenes strains selected on ciprofloxacin or the disinfectant benzalkonium chloride exhibit reduced susceptibility to ciprofloxacin, gentamicin, benzalkonium chloride, and other toxic compounds. Applied and environmental microbiology 77: 8714–8721. 10.1128/AEM.05941-11 22003016 PMC3233111

[44] Gadea R, Fuentes MÁF, Pulido RP, Gálvez A, Ortega E (2017a) Effects of exposure to quaternary-ammonium-based biocides on antimicrobial susceptibility and tolerance to physical stresses in bacteria from organic foods. Food microbiology 63: 58–71. 10.1016/j.fm.2016.10.037 28040182

[45] Coelho JR, Carriço JA, Knight D, Martínez J-L, Morrissey I, Oggioni MR, et al (2013) The Use of Machine Learning Methodologies to Analyse Antibiotic and Biocide Susceptibility in Staphylococcus aureus. PLOS ONE 8: e55582 10.1371/journal.pone.0055582 23431361 PMC3576404

[46] Lambert RJ, Joynson J, Forbes B (2001) The relationships and susceptibilities of some industrial, laboratory and clinical isolates of Pseudomonas aeruginosa to some antibiotics and biocides. J Appl Microbiol 91: 972–984. 11851804 10.1046/j.1365-2672.2001.01460.x

[47] Joynson JA, Forbes B, Lambert RJ (2002) Adaptive resistance to benzalkonium chloride, amikacin and tobramycin: the effect on susceptibility to other antimicrobials. J Appl Microbiol 93: 96–107. 12067378 10.1046/j.1365-2672.2002.01667.x

[48] Loughlin MF, Jones MV, Lambert PA (2002) Pseudomonas aeruginosa cells adapted to benzalkonium chloride show resistance to other membrane-active agents but not to clinically relevant antibiotics. Journal of Antimicrobial Chemotherapy 49: 631–639. 11909837 10.1093/jac/49.4.631

[49] Beier RC, Callaway TR, Andrews K, Poole TL, Crippen TL, Aderson RC, et al (2017) Disinfectant and antimicrobial susceptibility profiles of Salmonella strains from feedlot water-sprinkled cattle: hides and feces. J Food Chem Nanotechnol 3: 50–59.

[50] Beier RC, Anderson PN, Hume ME, Poole TL, Duke SE, Crippen TL, et al (2011) Characterization of Salmonella enterica isolates from turkeys in commercial processing plants for resistance to antibiotics, disinfectants, and a growth promoter. Foodborne pathogens and disease 8: 593–600. 10.1089/fpd.2010.0702 21235389

[51] Lambert RJ (2004) Comparative analysis of antibiotic and antimicrobial biocide susceptibility data in clinical isolates of methicillin-sensitive Staphylococcus aureus, methicillin-resistant Staphylococcus aureus and Pseudomonas aeruginosa between 1989 and 2000. J Appl Microbiol 97: 699–711. 10.1111/j.1365-2672.2004.02345.x 15357719

[52] CLSI Methods for Dilution Antimicrobial Susceptibility Tests for Bacteria That Grow Aerobically. 11th ed. CLSI standard M07 Wayne, PA: Clinical and Laboratory Standards Institute; 2018.

[53] CDC (2011) Multistate Outbreak of Human Salmonella Heidelberg Infections Linked to Ground Turkey

[54] Routh J, Pringle J, Mohr M, Bidol S, Arends K, Adams-Cameron M., et al (2015) Nationwide outbreak of multidrug-resistant Salmonella Heidelberg infections associated with ground turkey: United States, 2011. Epidemiology & Infection 143: 3227–3234. 25865382 10.1017/S0950268815000497 PMC9150975

[55] Frye JG, Jackson CR (2013) Genetic mechanisms of antimicrobial resistance identified in Salmonella enterica, Escherichia coli, and Enteroccocus spp. isolated from US food animals. Frontiers in microbiology 4: 135 10.3389/fmicb.2013.00135 23734150 PMC3661942

[56] Leader BT, Frye JG, Hu J, Fedorka-Cray PJ, Boyle DS (2009) High-throughput molecular determination of Salmonella enterica serovars by use of multiplex PCR and capillary electrophoresis analysis. Journal of clinical microbiology 47: 1290–1299. 10.1128/JCM.02095-08 19261787 PMC2681873

[57] Clinical and Laboratory Standards Institute 2015 Performance standards for antimicrobial susceptibility testing: 25th informational supplement (m100–S25) Clinical and Laboratory Standards Institute, Wayne, PA 17.

[58] Stevenson NW (2008) Reduced susceptibility of Salmonella enterica to biocides: ProQuest.

[59] Kautz M (2010) The effects of reduced susceptibility of Salmonella enterica to DTAC on some indicators of pathogenicity: University of Delaware.

[60] Kautz MJM, Dvorzhinskiy A, Frye JG, Stevenson N, Herson DS (2013) Pathogenicity of dodecyltrimethylammonium chloride-resistant Salmonella enterica. Applied and environmental microbiology 79: 2371–2376. 10.1128/AEM.03228-12 23377943 PMC3623252

[61] Parkhill J, Dougan G, James K, Thomson N, Pickard D, Wain J, et al (2001) Complete genome sequence of a multiple drug resistant Salmonella enterica serovar Typhi CT18. Nature 413: 848–852. 10.1038/35101607 11677608

[62] McClelland M, Sanderson KE, Spieth J, Clifton SW, Latreille P, Courtney L, et al (2001) Complete genome sequence of Salmonella enterica serovar Typhimurium LT2. Nature 413: 852–856. 10.1038/35101614 11677609

[63] Frye JG, Jesse T, Long F, Rondeau G, Porwollik S, McClelland M, et al (2006) DNA microarray detection of antimicrobial resistance genes in diverse bacteria. International journal of antimicrobial agents 27: 138–151. 10.1016/j.ijantimicag.2005.09.021 16427254

[64] Wang H, Wang H, Xing T, Wu N, Xu X, Malakaret K., et al (2016) Removal of Salmonella biofilm formed under meat processing environment by surfactant in combination with bio-enzyme. LWT-Food Science and Technology 66: 298–304.

[65] Mah T-FC, O'Toole GA (2001) Mechanisms of biofilm resistance to antimicrobial agents. Trends in Microbiology 9: 34–39. 11166241 10.1016/s0966-842x(00)01913-2

[66] Beier R, Bischoff K, Ziprin R, Poole T, Nisbet D (2005) Chlorhexidine susceptibility, virulence factors, and antibiotic resistance of beta-hemolytic Escherichia coli isolated from neonatal swine with diarrhea. Bulletin of environmental contamination and toxicology 75: 835–844. 10.1007/s00128-005-0826-5 16400568

[67] Russell AD (2000) Do Biocides Select for Antibiotic Resistance?*. Journal of Pharmacy and Pharmacology 52: 227–233. 10714955 10.1211/0022357001773742

[68] Leung P, Boost MV, Cho P (2004) Effect of storage temperatures and time on the efficacy of multipurpose solutions for contact lenses. Ophthalmic and Physiological Optics 24: 218–224. 10.1111/j.1475-1313.2004.00189.x 15130170

[69] Russell A (1996) Activity of biocides against mycobacteria. Journal of applied bacteriology 81. 8972124

[70] Gilbert P, McBain AJ (2003) Potential impact of increased use of biocides in consumer products on prevalence of antibiotic resistance. Clinical microbiology reviews 16: 189–208. 10.1128/CMR.16.2.189-208.2003 12692093 PMC153147

[71] Bloomfield SF (2002) Significance of biocide usage and antimicrobial resistance in domiciliary environments. Journal of Applied Microbiology 92: 144S–157S. 12000623

[72] Beumer R, Bloomfield S, Exner M, Fara G, Nath K, Scott E. Microbial resistance and biocides: A review by the International Forum on Home Hygiene (IFH); 2000 International Scientific Forum on Home Hygiene.

[73] Maillard JY (2002) Bacterial target sites for biocide action. Journal of applied microbiology 92. 12000609

[74] Jorgensen J, Turnidge J, Washington J, Murray P, Pfaller M, Tenover FC, et al (1999) Antibacterial susceptibility tests: dilution and disk diffusion Methods Manual of Clinical Microbiology.) Geo. F. Brooks Publisher, Washington.

[75] Reller LB, Weinstein M, Jorgensen JH, Ferraro MJ (2009) Antimicrobial susceptibility testing: a review of general principles and contemporary practices. Clinical Infectious Diseases 49: 1749–1755. 10.1086/647952 19857164

[76] FDA (2014) US Food and Drug Administration. "CFR-code of federal regulations title 21." Current good manufacturing practice for finished pharmaceuticals Part 211 (2014). https://wwwaccessdatafdagov/scripts/cdrh/cfdocs/cfcfr/CFRSearchcfm?fr=1781010.

[77] McDonnell G, Russell AD (1999) Antiseptics and Disinfectants: Activity, Action, and Resistance. Clinical Microbiology Reviews 12: 147–179. 9880479 10.1128/cmr.12.1.147 PMC88911

[78] Tezel U, Pavlostathis SG (2015) Quaternary ammonium disinfectants: microbial adaptation, degradation and ecology. Curr Opin Biotechnol 33: 296–304. 10.1016/j.copbio.2015.03.018 25864173

[79] FDA (2004) Food and Drug Administration Department of Health and Human Services 21 CFR Part 173 375. Federal Register/Vol 69 No 64/Friday April 2 2004/Rules and Regulations.

[80] Cortesia C, Vilchèze C, Bernut A, Contreras W, Gómez K, de Waard J, et al (2014) Acetic acid, the active component of vinegar, is an effective tuberculocidal disinfectant. MBio 5: e00013–00014. 10.1128/mBio.00013-14 24570366 PMC3940030

[81] Tezel U, Pavlostathis SG (2011) Role of quaternary ammonium compounds on antimicrobial resistance in the environment. Antimicrobial resistance in the environment: 349–387.

[82] Kong Y-J, Park B-K, Oh D-H (2001) Antimicrobial activity of Quercus mongolica leaf ethanol extract and organic acids against food-borne microorganisms. Korean Journal of Food Science and Technology 33: 178–183.

[83] Mani-Lopez E, García HS, López-Malo A (2012) Organic acids as antimicrobials to control Salmonella in meat and poultry products. Food Research International 45: 713–721.

[84] Byrd J, Hargis B, Caldwell D, Bailey R, Herron K, McReynalds JL, et al (2001) Effect of lactic acid administration in the drinking water during preslaughter feed withdrawal on Salmonella and Campylobacter contamination of broilers. Poultry science 80: 278–283. 10.1093/ps/80.3.278 11261556

[85] Van Immerseel F, Russell J, Flythe M, Gantois I, Timbermont L, Pasmans F, et al (2006) The use of organic acids to combat Salmonella in poultry: a mechanistic explanation of the efficacy. Avian Pathology 35: 182–188. 10.1080/03079450600711045 16753609

[86] Khan SH, Iqbal J (2016) Recent advances in the role of organic acids in poultry nutrition. Journal of Applied Animal Research 44: 359–369.

[87] FSIS (2012) Safe and suitable ingredients used in the production of meat, poultry, and egg products FSIS Notice 49–94.

[88] Kotula KL, Thelappurate R (1994) Microbiological and sensory attributes of retail cuts of beef treated with acetic and lactic acid solutions. Journal of Food Protection 57: 665–670. 10.4315/0362-028X-57.8.665 31121755

[89] Smulders F, Greer G (1998) Integrating microbial decontamination with organic acids in HACCP programmes for muscle foods: prospects and controversies. International Journal of Food Microbiology 44: 149–169. 9851597 10.1016/s0168-1605(98)00123-8

[90] Wales A, McLaren I, Rabie A, Gosling RJ, Martelli F, Sayers R, et al (2013) Assessment of the anti-Salmonella activity of commercial formulations of organic acid products. Avian Pathology 42: 268–275. 10.1080/03079457.2013.782097 23600468

[91] Rao MV (2007) Acidified Sodium Chlorite (ASC) Chemical and Technical Assessment.

[92] Warf C, Kemp G. The chemistry and mode of action of acidified sodium chlorite; 2001. pp. 1–91.

[93] Wang H, Ye K, Xu X, Zhou G (2014) Optimization of an acidified sodium chlorite solution for reducing pathogenic bacteria and maintaining sensory characteristics of poultry meat in simulation slaughter process. Journal of food processing and preservation 38: 397–405.

[94] Inatsu Y, Bari ML, Kawasaki S, Isshiki K, Kawamoto S (2005) Efficacy of acidified sodium chlorite treatments in reducing Escherichia coli O157:H7 on Chinese cabbage. J Food Prot 68: 251–255. 15726965 10.4315/0362-028x-68.2.251

[95] Castillo A, Lucia LM, Kemp GK, Acuff GR (1999) Reduction of Escherichia coli O157:H7 and Salmonella typhimurium on beef carcass surfaces using acidified sodium chlorite. J Food Prot 62: 580–584. 10382644 10.4315/0362-028x-62.6.580

[96] Ransom J, Belk K, Sofos J, Stopforth J, Scanga J, Smith GC. (2003) Comparison of intervention technologies for reducing Escherichia coli O157: H7 on beef cuts and trimmings. Food Protection Trends 23: 24–34.

[97] Kalchayanand N, Arthur TM, Bosilevac JM, Schmidt JW, Wang R, Shalkelford SD, et al (2012) Evaluation of commonly used antimicrobial interventions for fresh beef inoculated with Shiga toxin–producing Escherichia coli serotypes O26, O45, O103, O111, O121, O145, and O157: H7. Journal of food protection 75: 1207–1212. 10.4315/0362-028X.JFP-11-531 22980002

[98] Kang JH, Jang YJ, Kim DJ, Park JW (2015) Antimicrobial effectiveness of cetylpyridinium chloride and zinc chloride-containing mouthrinses on bacteria of halitosis and peri-implant disease. Int J Oral Maxillofac Implants 30: 1341–1347. 10.11607/jomi.3824 26478974

[99] Trachoo N, Frank JF (2002) Effectiveness of chemical sanitizers against Campylobacter jejuni–containing biofilms. Journal of food protection 65: 1117–1121. 12117244 10.4315/0362-028x-65.7.1117

[100] Nde CW, Jang H-J, Toghrol F, Bentley WE (2009) Global transcriptomic response of Pseudomonas aeruginosa to chlorhexidine diacetate. Environmental science & technology 43: 8406–8415. 19924977 10.1021/es9015475

[101] Kampf G, Kramer A (2004) Epidemiologic background of hand hygiene and evaluation of the most important agents for scrubs and rubs. Clin Microbiol Rev 17: 863–893. 10.1128/CMR.17.4.863-893.2004 15489352 PMC523567

[102] Wei C-I, Cook DL, Kirk JR (1985) Use of chlorine compounds in the food industry. Food Technology.

[103] Rutala WA, Weber DJ (2008) Guideline for disinfection and sterilization in healthcare facilities, 2008: Centers for Disease Control (US).

[104] Kalchayanand N, Arthur TM, Bosilevac JM, Brichta-Harhay DM, Guerini MN, Shakelford SD, et al (2009) Effectiveness of 1, 3-Dibromo-5, 5 Dimethylhydantoin on Reduction of Escherichia coli O157: H7–and Salmonella-Inoculated Fresh Meat. Journal of food protection 72: 151–156. 19205477 10.4315/0362-028x-72.1.151

[105] USDA (2012) USDA. 2012 Safe and Suitable Ingredients Used in the Production of Meat, Poultry, and Egg Products Food Safety Inspection Service, USDA Washington, D.C.

[106] FDA U (2006) Secondary direct food additives permitted in food and human consumption. 21CFR173.310. Fed. Regist. 3:138–140.

[107] Sharma CS, Williams SK, Schneider KR, Schmidt RH, Rodrick GE (2013) Mechanism of antimicrobial action of sodium metasilicate against Salmonella enterica serovar Typhimurium. Foodborne Pathog Dis 10: 995–1001. 10.1089/fpd.2013.1556 23980709

[108] Huang H (2010) The Effects of Sodium Metasilicate on Antimicrobial, Sensory, Physical and Chemical Characteristics of Fresh Commercial Chicken Breast Meat Stored at Four Degrees Celsius for Nine Days. 10.3382/ps.2010-01227 21489963

[109] Sharma C, Williams S, Schneider K, Schmidt R, Rodrick G (2012) Sodium metasilicate affects growth of Salmonella Typhimurium in fresh, boneless, uncooked chicken breast fillets stored at 4° C for 7 days. Poultry science 91: 719–723. 10.3382/ps.2011-01848 22334748

[110] Register Federal (1994) Use of trisodium phosphate on raw, chilled poultry carcasses. Fed. Regist. 59:551–554.

[111] FSIS U (2017) 7120.1. Rev. 41. 5/12/17. Safe and Suitable Ingredients used in the Production of Meat, Poultry, And Egg Products. FSIS Directive.

[112] Keener K, Bashor M, Curtis P, Sheldon B, Kathariou S (2004) Comprehensive review of Campylobacter and poultry processing. Comprehensive reviews in food science and food safety 3: 105–116. 10.1111/j.1541-4337.2004.tb00060.x 33430546

[113] González RC, Fernández MdCG, Moreno B, Calleja CA (2002) Trisodium phosphate (TSP) treatment for decontamination of poultry: review. Food science and technology international = Ciencia y tecnología de alimentos internacional 8: 11–24.

[114] Sarjit A, Dykes GA (2015) Trisodium phosphate and sodium hypochlorite are more effective as antimicrobials against Campylobacter and Salmonella on duck as compared to chicken meat. International Journal of Food Microbiology 203: 63–69. 10.1016/j.ijfoodmicro.2015.02.026 25791251

[115] Selvi MS, Sasikumar S, Gomathi S, Rajkumar P, Sasikumar P, Sadasivam SG. (2014) Isolation and characterization of arsenic resistant bacteria from agricultural soil, and their potential for arsenic bioremediation. International Journal of Agricultural Policy and Research 2: 393–405.

[116] Ratnaike RN (2003) Acute and chronic arsenic toxicity. Postgraduate medical journal 79: 391–396. 10.1136/pmj.79.933.391 12897217 PMC1742758

[117] Kowalski LM, Reid WM (1975) Effects of roxarsone on pigmentation and coccidiosis in broilers. Poultry science 54: 1544–1549. 10.3382/ps.0541544 1187515

[118] Mellon MG, Benbrook C, Benbrook KL (2001) Hogging it: estimates of antimicrobial abuse in livestock: Union of Concerned Scientists.

[119] Chapman H, Johnson Z (2002) Use of antibiotics and roxarsone in broiler chickens in the USA: analysis for the years 1995 to 2000. Poultry Science 81: 356–364. 10.1093/ps/81.3.356 11902412

[120] FDA (2015a) Product Safety Information. Questions and Answers Regarding 3-Nitro (Roxarsone). Available: http://wwwfdagov/AnimalVeterinary/SafetyHealth/ProductSafetyInformation/ucm258313htm.

[121] FDA (2015b) FDA Announces Pending Withdrawal of Approval of Nitarsone.

[122] Rutherford D, Bednar A, Garbarino J, Needham R, Staver K, Wershaw RL. (2003) Environmental fate of roxarsone in poultry litter. Part II. Mobility of arsenic in soils amended with poultry litter. Environmental science & technology 37: 1515–1520. 12731832 10.1021/es026222+

[123] Garbarino J, Bednar A, Rutherford D, Beyer R, Wershaw R (2003) Environmental fate of roxarsone in poultry litter. I. Degradation of roxarsone during composting. Environmental Science & Technology 37: 1509–1514. 12731831 10.1021/es026219q

[124] Bednar A, Garbarino J, Ferrer I, Rutherford D, Wershaw R, Wershaw RL. (2003) Photodegradation of roxarsone in poultry litter leachates. Science of The Total Environment 302: 237–245. 12526912 10.1016/s0048-9697(02)00322-4

[125] Sapkota AR, Price LB, Silbergeld EK, Schwab KJ (2006) Arsenic Resistance in Campylobacter spp. Isolated from Retail Poultry Products. Applied and Environmental Microbiology 72: 3069–3071. 10.1128/AEM.72.4.3069-3071.2006 16598022 PMC1449025

[126] Yang H-C, Rosen BP (2016) New mechanisms of bacterial arsenic resistance. Biomedical Journal 39: 5–13. 10.1016/j.bj.2015.08.003 27105594 PMC6138428

[127] Silver S, Phung LT (1996) Bacterial heavy metal resistance: new surprises. Annual Reviews in Microbiology 50: 753–789. 10.1146/annurev.micro.50.1.753 8905098

