# Supplementary material for: An assay for determining the susceptibility of Salmonella isolates to commercial and household biocides
Source: PLoS One. 2018 Dec 20;13(12):e0209072. doi: 10.1371/journal.pone.0209072 (PMC6301668; doi:10.1371/journal.pone.0209072)
Supplement: S1 Table — (DOCX) [file pone.0209072.s001.docx]

**Supporting Information**

**S1 Table**. Minimum inhibitory concentration (MIC) of 17 disinfecting chemicals / biocides in µg ml^-1^ for each isolates with Antibiotic Resistance (AR), source and year of Isolation.

| Isolates | Serotype | source | **AR** | DC 32-512 or 64-1024 µg ml^-1^ | CPC 10-160 µg ml^-1^ | BKC 5-80 µg ml^-1^ | HB 10-160 or 20-320 µg ml^-1^ | HC 10-160 or 20-320 µg ml^-1^ | AA 410-6560 µg ml^-1^ | LA 944-15104 µg ml^-1^ | CA 789-12624 µg ml^-1^ | PXA 220-3520 µg ml^-1^ | ASC 80-1280 µg ml^-1^ | CHX 0.75 -48 µg ml^-1^ | DBH 156-2496 or 228-3648 µg ml^-1^ | SHB 394-6304 µg ml^-1^ | SM 7540-120640 µg ml^-1^ | TSP 4714-75424 µg ml^-1^ | ARA 52-6656 or 26-3328 µg ml^-1^ | ARI 7-896 µg ml^-1^ |
| --- | --- | --- | --- | --- | --- | --- | --- | --- | --- | --- | --- | --- | --- | --- | --- | --- | --- | --- | --- | --- |
| CT 18 | Typhi | ATCC |  | 256 | 40 | 40 | 80 | 80 | 3280 | 7552 | **12624** | 1760 | 160 | **12** | 1824 | 6304 | 60320 | 37712 | **3328** | 56 |
| LT2 | Typhimurium | ATCC |  | 256 | 40 | 40 | 160 | 80 | 3280 | 7552 | **12624** | 1760 | 320 | **12** | 1824 | 6304 | 60320 | 37712 | **3328** | 112 |
| 14028 | Typhimurium | ATCC |  | 256 | 40 | 40 | 160 | 40 | 3280 | 7552 | 6312 | 1760 | 320 | **12** | 912 | 6304 | 60320 | 18856 | **3328** | 112 |
| SE | Enteritidis | U. of Del. |  | 128 | 40 | 40 | 80 | 80 | 1640 | 7552 | 6312 | 1760 | 320 | 3 | 1824 | 6304 | 60320 | 18856 | 416 | 56 |
| L1 | Enteritidis | U. of Del. |  | **>512** | 20 | 20 | 80 | 40 | 1640 | 3776 | 3156 | 880 | 320 | **24** | 1824 | 6304 | 60320 | 18856 | 208 | 28 |
| L2 | Enteritidis | U. of Del. |  | 512 | 20 | 20 | 80 | 40 | 1640 | 3776 | 3156 | 880 | 80 | **12** | 1824 | 6304 | 60320 | 9428 | 208 | 28 |
| L3 | Enteritidis | U. of Del. |  | **>512** | 20 | 10 | 80 | 80 | 1640 | 3776 | 3156 | 880 | 160 | **24** | 1824 | 6304 | 60320 | 18856 | 416 | 28 |
| BA1001 | Kentucky | Young chicken | 2 | 256 | 40 | 40 | 80 | 80 | 1640 | 3776 | 3156 | 880 | 640 | 3 | 2496 | 6304 | 60320 | 37712 | 1664 | 56 |
| BA1002 | Heidelberg | Young chicken | 5 | 256 | 20 | 40 | 80 | 80 | 1640 | 3776 | 1578 | 880 | 640 | 1.5 | 2496 | 6304 | 120640 | 37712 | 416 | 56 |
| BA1003 | Heidelberg | Swine | 7 | 256 | 80 | 40 | 160 | 160 | 3280 | 7552 | **12624** | 1760 | 160 | 6 | 3648 | 3152 | 60320 | 37712 | 416 | 56 |
| BA1004 | Heidelberg | Cattle | 13 | 256 | 80 | 40 | 160 | 160 | 3280 | 7552 | **12624** | 1760 | 320 | 6 | 3648 | 3152 | 60320 | 37712 | 208 | 56 |
| BA1005 | Newport | Cow/bull | 9 | 256 | 80 | 40 | 160 | 160 | 3280 | 7552 | 6312 | 1760 | 640 | **12** | 3648 | 3152 | 60320 | 37712 | **>3328** | 112 |
| BA1006 | Newport | Horse | 10 | 256 | 80 | 40 | 160 | 160 | 3280 | 7552 | **12624** | 1760 | 640 | **12** | 3648 | 6304 | 60320 | 37712 | **>3328** | 112 |
| BA1007 | Newport | Ground beef | 9 | 256 | 80 | 40 | 160 | 160 | 1640 | 7552 | **12624** | 1760 | 640 | **12** | 3648 | 3152 | 60320 | 37712 | **>3328** | 112 |
| BA1008 | Newport | Ground beef | 9 | 256 | 80 | 40 | 160 | 160 | 3280 | 7552 | **12624** | 1760 | 320 | **12** | 3648 | 6304 | 60320 | 37712 | **>3328** | 112 |
| BA1009 | Newport | Cattle | 10 | 256 | 80 | 40 | 160 | 160 | 3280 | 7552 | **12624** | 1760 | 640 | **12** | 3648 | 6304 | 60320 | 37712 | **>3328** | 112 |
| BA1010 | Newport | Dairy cattle | 11 | 256 | 80 | 40 | 160 | 160 | 3280 | 7552 | **12624** | 1760 | **1280** | **12** | 3648 | 6304 | 60320 | 37712 | **>3328** | 112 |
| BA1011 | Newport | Dog | 12 | 256 | 80 | 40 | 160 | 160 | 3280 | 7552 | **12624** | 1760 | 320 | **12** | 3648 | 3152 | 60320 | 37712 | **3328** | 112 |
| BA1012 | Newport | Cattle | 9 | 256 | 80 | 40 | 160 | 160 | 3280 | 7552 | **12624** | 1760 | 640 | **12** | 3648 | 6304 | 60320 | 37712 | **3328** | 56 |
| BA1013 | Newport | Environmental | 13 | 128 | 80 | 40 | 80 | 80 | 1640 | 7552 | 6312 | 880 | 640 | 3 | 2496 | 6304 | 60320 | 37712 | 416 | 28 |
| BA1014 | Typhimurium* | Ground beef | 5 | 256 | 80 | 40 | 160 | 160 | 3280 | 7552 | **12624** | 1760 | 640 | **12** | 3648 | 3152 | 60320 | 37712 | **3328** | 56 |
| BA1015 | Typhimurium* | Swine | 5 | 256 | 80 | 40 | 160 | 160 | 3280 | 7552 | **12624** | 1760 | 320 | **12** | 3648 | 3152 | 60320 | 37712 | **>3328** | 112 |
| BA1016 | Typhimurium* | Cat | 4 | 256 | 80 | 40 | 80 | 80 | 1640 | 7552 | 6312 | 880 | 640 | 1.5 | 2496 | 6304 | 60320 | 37712 | 1664 | 56 |
| BA1017 | Typhimurium* | Young chicken | 7 | 256 | 80 | 40 | 160 | 160 | 1640 | 7552 | **12624** | 1760 | 640 | **12** | 3648 | 6304 | 60320 | 37712 | 1664 | 56 |
| BA1018 | Typhimurium* | Market hog | 2 | 128 | 80 | 40 | 80 | 80 | 1640 | 3776 | 3156 | 880 | 160 | 1.5 | 2496 | 6304 | 60320 | 18856 | 832 | 56 |
| BA1019 | Typhimurium* | Young chicken | 2 | 256 | 40 | 40 | 80 | 80 | 1640 | 3776 | 3156 | 880 | **>640** | 1.5 | 2496 | 6304 | 60320 | 37712 | 1664 | 56 |
| BA1020 | Typhimurium* | Turkey | 5 | 256 | 80 | 40 | 160 | 160 | 1640 | 7552 | **12624** | 1760 | **1280** | **12** | 3648 | 6304 | 60320 | 37712 | 416 | 56 |
| BA1021 | Typhimurium | Turkey | 4 | 256 | 80 | 40 | 160 | 160 | 3280 | 7552 | **12624** | 1760 | **1280** | **12** | 3648 | 6304 | 60320 | 37712 | **3328** | 112 |
| BA1022 | Typhimurium | Swine | 5 | 256 | 80 | 40 | 160 | 160 | 3280 | 7552 | **12624** | 1760 | 640 | **12** | 3648 | 6304 | 60320 | 37712 | 1664 | 56 |
| BA1023 | Agona | Cattle | 11 | 256 | 80 | 40 | 160 | 80 | 3280 | 7552 | 6312 | 1760 | 320 | **12** | 1824 | 6304 | 60320 | 37712 | 1664 | 56 |
| BA1024 | Agona | Cattle | 11 | 256 | 80 | 40 | 160 | 160 | 3280 | 7552 | **12624** | 1760 | **1280** | **12** | 3648 | 6304 | 60320 | 37712 | **3328** | 112 |
| BA1025 | Agona | Swine | 11 | 512 | 80 | 40 | **>160** | 160 | 1640 | 7552 | **12624** | 1760 | 640 | **12** | 3648 | 6304 | 60320 | 37712 | 1664 | 56 |
| BA1026 | Agona | Ground beef | 7 | 256 | 80 | 40 | 160 | 160 | 3280 | 7552 | **12624** | 1760 | 640 | **12** | 3648 | 6304 | 60320 | 37712 | 1664 | 56 |
| BA1027 | Dublin | Chicken | 10 | 256 | 40 | 40 | 160 | 80 | 1640 | 7552 | 6312 | 880 | 160 | **12** | 3648 | 6304 | 60320 | 37712 | 416 | 28 |
| BA1028 | Dublin | Ground beef | 5 | 256 | 40 | 40 | 80 | 80 | 1640 | 3776 | 3156 | 880 | 320 | 6 | 2496 | 3152 | 60320 | 18856 | 416 | 28 |
| BA1029 | Dublin | Ground beef | 6 | 256 | 40 | 40 | 80 | 80 | 1640 | 3776 | 3156 | 880 | 640 | 3 | 2496 | 3152 | 60320 | 37712 | 416 | 28 |
| BA1030 | Dublin | Cattle | 6 | 256 | 40 | 40 | 80 | 80 | 1640 | 3776 | 3156 | 880 | 640 | **12** | 2496 | 3152 | 30160 | 37712 | 208 | 14 |
| BA1031 | Dublin | Dairy cattle | 10 | 256 | 40 | 40 | 160 | 80 | 1640 | 7552 | 6312 | 1760 | 160 | **12** | 3648 | 6304 | 60320 | 37712 | 416 | 28 |
| BA1032 | Dublin | Steer/heifer | 7 | 256 | 80 | 40 | 80 | 160 | 1640 | 7552 | 6312 | 1760 | 160 | **12** | 3648 | 6304 | 60320 | 37712 | 416 | 14 |
| BA1033 | Dublin | Ground beef | 7 | 128 | 40 | 20 | 80 | 80 | 1640 | 3776 | 3156 | 880 | 320 | 3 | 1248 | 3152 | 60320 | 37712 | 416 | 28 |
| BA1034 | Uganda | Horse | 11 | 256 | 80 | 40 | 80 | 80 | 1640 | 3776 | 3156 | 880 | 160 | 3 | 1248 | 788 | 60320 | 37712 | 416 | 28 |
| BA1035 | Uganda | Environmental | 11 | 256 | 40 | 20 | 80 | 80 | 1640 | 3776 | 3156 | 880 | 160 | 1.5 | 1248 | 788 | 30160 | 37712 | 208 | 7 |
| BA1036 | Uganda | Cattle | 12 | 256 | 80 | 40 | 160 | 80 | 1640 | 3776 | 6312 | 880 | 160 | 3 | 624 | 788 | 60320 | 37712 | 208 | 7 |
| BA1037 | Uganda | Ground beef | 10 | 256 | 40 | 40 | 160 | 80 | 1640 | 7552 | 3156 | 880 | 320 | 1.5 | 1248 | 1576 | 60320 | 37712 | 208 | 7 |
| BA1038 | Thompson | Young chicken | 5 | 256 | 80 | 40 | 80 | 80 | 1640 | 7552 | 3156 | 440 | 640 | 1.5 | 1248 | 1576 | 60320 | 37712 | 832 | 56 |
| BA1039 | Worthington | Young chicken | 5 | 128 | 80 | 40 | 80 | 80 | 1640 | 7552 | 3156 | 880 | 320 | 3 | 1248 | 788 | 60320 | 37712 | **>6656** | **>896** |
| BA1040 | Adelaide | Steer/Heifer | 9 | 256 | 80 | 40 | 80 | 80 | 1640 | 3776 | 3156 | 880 | 160 | 3 | 1248 | 1576 | 60320 | 37712 | 416 | 28 |
| BA1041 | I 9,12:-:- | Cow/bull | 5 | 128 | 40 | 20 | 40 | 80 | 1640 | 3776 | 1578 | 880 | 320 | 3 | 312 | 6304 | 60320 | 37712 | 104 | 7 |
| BA1042 | I 9,12:-:- | Ground beef | 11 | 256 | 80 | 40 | 80 | 80 | 1640 | 3776 | 3156 | 880 | 320 | 3 | 624 | 6304 | 60320 | 37712 | 208 | 14 |
| BA1043 | I 9,12:-:- | Dairy cattle | 11 | 256 | 80 | 40 | 80 | 80 | 1640 | 3776 | 3156 | 880 | 160 | 3 | 624 | 788 | 60320 | 37712 | 208 | 14 |
| BA1044 | I 9,12:-:- | Ground beef | 10 | 256 | 80 | 40 | 80 | 80 | 1640 | 3776 | 3156 | 880 | 160 | 3 | 624 | - | 60320 | 37712 | 208 | 14 |
| BA1045 | I 9,12:-:- | Ground beef | 9 | 256 | 40 | 40 | 80 | 80 | 1640 | 3776 | 1578 | 880 | 320 | 3 | 624 | - | 60320 | 37712 | 208 | 14 |
| BA1046 | I 9,12:-:- | Cattle | 7 | 256 | 80 | 40 | 80 | 80 | 1640 | 3776 | 3156 | 440 | 160 | 1.5 | 1248 | 788 | 60320 | 37712 | 208 | 14 |
| BA1047 | I 9,12:-:- | Dairy cattle | 11 | 256 | 80 | 20 | 80 | 80 | 1640 | 3776 | 3156 | 440 | 80 | ≤0.75 | 624 | - | 30160 | 37712 | 104 | 7 |
| BA1048 | I 9,12:-:- | Ground beef | 7 | 256 | 80 | 40 | 160 | 160 | 1640 | 3776 | 3156 | 880 | 320 | 6 | 624 | - | 30160 | 37712 | 52 | 7 |
| BA1049 | Alachua | Ground turkey | 10 | 256 | 80 | 40 | 80 | 80 | 1640 | 3776 | 3156 | 880 | 160 | 3 | 1248 | 788 | 60320 | 37712 | **6656** | **448** |
| BA1050 | Alachua | Turkey Swab | 10 | 256 | 80 | 40 | 80 | 80 | 1640 | 3776 | 3156 | 880 | **1280** | 3 | 1248 | 788 | 60320 | 37712 | **>6656** | **896** |
| BA1051 | Orion | Young chicken | 6 | 256 | 160 | 40 | 80 | 160 | 1640 | 3776 | 3156 | 880 | 640 | 3 | 1248 | 788 | 60320 | 37712 | **>6656** | **>896** |
| BA1052 | Orion | Ground turkey | 7 | 256 | 80 | 40 | 160 | 80 | 1640 | 3776 | 3156 | 880 | 320 | 3 | 624 | 788 | 60320 | 37712 | 208 | 28 |
| BA1053 | Krefeld | Swine | 10 | 256 | 40 | 40 | 80 | 80 | 1640 | 3776 | 3156 | 880 | 320 | ≤0.75 | 1248 | 1576 | 60320 | 37712 | 1664 | 56 |
| BA1054 | Krefeld | Swine | 6 | 256 | 40 | 40 | 80 | 80 | 1640 | 3776 | 3156 | 440 | 320 | ≤0.75 | 624 | 788 | 60320 | 37712 | 1664 | 56 |
| BA1055 | Krefeld | Market hog | 6 | 128 | 80 | 40 | 80 | 80 | 1640 | 7552 | 3156 | 880 | 320 | ≤0.75 | 1248 | 1576 | 60320 | 37712 | 1664 | 56 |
| BA1056 | Krefeld | Swine | 8 | 256 | 80 | 40 | 80 | 80 | 820 | 3776 | 6312 | 880 | 320 | ≤0.75 | 624 | 1576 | 120640 | 37712 | 1664 | 56 |
| BA1057 | Krefeld | Swine | 10 | 256 | 80 | 40 | 160 | 160 | 1640 | 7552 | 6312 | 880 | 640 | 1.5 | 624 | 1576 | 120640 | 37712 | 1664 | 56 |
| BA1058 | Krefeld | Market hog | 6 | 256 | 40 | 40 | 160 | 80 | 1640 | 7552 | 6312 | 880 | 320 | ≤0.75 | 624 | 1576 | 120640 | 37712 | 1664 | 56 |
| BA1059 | Krefeld | Swine | 7 | 256 | 80 | 40 | 80 | 80 | 1640 | 3776 | 3156 | 880 | 320 | 0.75 | 1248 | 788 | 60320 | 37712 | 1664 | 56 |
| BA1060 | Krefeld | Swine | 10 | 128 | 80 | 40 | 160 | 80 | 1640 | 3776 | 3156 | 440 | 320 | ≤0.75 | 1248 | 788 | 60320 | 37712 | 832 | 56 |
| BA1061 | Krefeld | Swine | 10 | 256 | 80 | 40 | 80 | 80 | 1640 | 7552 | 6312 | 440 | 320 | 3 | 624 | 1576 | 120640 | 37712 | 832 | 56 |
| BA1062 | Bardo | Dairy cattle | 10 | 256 | 80 | 40 | 80 | 80 | 1640 | 3776 | 3156 | 880 | 320 | 1.5 | 1248 | 788 | 120640 | 37712 | 832 | 56 |
| BA1063 | Bardo | Ground beef | 9 | 256 | 80 | 40 | 80 | 80 | 1640 | 3776 | 3156 | 880 | 640 | 3 | 1248 | 788 | 60320 | 37712 | 832 | 56 |
| BA1064 | Bardo | Dairy cattle | 10 | 256 | 80 | 40 | 80 | 80 | 1640 | 3776 | 3156 | 880 | 320 | 0.75 | 624 | 788 | 60320 | 37712 | 832 | 56 |
| BA1065 | Bardo | Dog | 10 | 256 | 80 | 40 | 80 | 80 | 1640 | 3776 | 3156 | 880 | **1280** | 3 | 1248 | 788 | 60320 | 37712 | 416 | 28 |
| BA1066 | Bardo | Horse | 11 | 128 | 40 | 40 | 160 | 160 | 3280 | 7552 | 6312 | 880 | 640 | 3 | 2496 | 6304 | 120640 | 37712 | 1664 | 56 |
| BA1067 | Bardo | Dairy cattle | 9 | 256 | 40 | 40 | 80 | 80 | 1640 | 7552 | 3156 | 880 | 320 | 1.5 | 1248 | 788 | 60320 | 37712 | 1664 | 56 |
| BA1068 | I 4,12:r:- | Cat | 5 | 256 | 80 | 40 | 80 | 80 | 1640 | 3776 | 3156 | 880 | 320 | 0.75 | 1248 | 788 | 60320 | 37712 | 416 | 28 |
| BA1069 | I 4,12:r:- | Young chicken | 5 | ≤64 | 80 | 40 | 80 | 80 | 1640 | 7552 | 3156 | 440 | 160 | 0.75 | 624 | 788 | 30160 | 18856 | 208 | 14 |
| BA1070 | I 4,12:r:- | Young chicken | 7 | 256 | 80 | 40 | 80 | 80 | 1640 | 7552 | 6312 | 880 | 320 | ≤0.75 | 624 | 1576 | 120640 | 18856 | 416 | 28 |
| BA1071 | I 4,12:r:- | Ground turkey | 10 | 128 | 80 | 40 | 80 | 80 | 3280 | 7552 | 6312 | 880 | 320 | ≤0.75 | 624 | 788 | 120640 | 37712 | 416 | 28 |
| BA1072 | I 4,12:-:- | Ground beef | 11 | 256 | 80 | 40 | 80 | 80 | 1640 | 3776 | 3156 | 880 | 320 | 1.5 | 1248 | 788 | 60320 | 37712 | 416 | 28 |
| BA1073 | I 4,12:-:- | Horse | 11 | 256 | 80 | 40 | 160 | 80 | 1640 | 3776 | 3156 | 440 | 320 | ≤0.75 | 1248 | 788 | 60320 | 37712 | 416 | 28 |
| BA1074 | I 4,12:-:- | Young chicken | 7 | 256 | 80 | 40 | 80 | 80 | 1640 | 3776 | 3156 | 880 | 640 | 3 | 624 | 788 | 60320 | 37712 | 832 | 56 |
| BA1075 | I 4,12:-:- | Young chicken | 7 | 128 | 80 | 40 | 80 | 80 | 1640 | 3776 | 3156 | 440 | 320 | 0.75 | 1248 | 788 | 60320 | 37712 | 208 | 28 |
| BA1076 | Putten | Swine | 6 | 256 | 80 | 40 | 80 | 80 | 1640 | 3776 | 3156 | 880 | 320 | 3 | 1248 | 788 | 120640 | 37712 | 832 | 56 |
| BA1077 | Heidelberg | Young Chicken | 5 | 256 | 80 | 40 | 160 | 160 | 1640 | 3776 | 3156 | 880 | 640 | 3 | 2496 | 6304 | 120640 | 37712 | 832 | 28 |
| BA1078 | Heidelberg | Ground Turkey | 3 | 256 | 80 | 40 | 160 | 80 | 1640 | 3776 | 6312 | 880 | 640 | 1.5 | 2496 | 6304 | 120640 | 37712 | 832 | 56 |
| BA1079 | Heidelberg | Ground Turkey | 9 | 256 | 80 | 40 | 160 | 160 | 1640 | 7552 | 6312 | 880 | 320 | 1.5 | 2496 | 6304 | 120640 | 37712 | 832 | 28 |
| BA1080 | Heidelberg | Human** | 4 | 256 | 80 | 40 | 160 | 80 | 1640 | 7552 | 6312 | 880 | 640 | 1.5 | 2496 | 6304 | 60320 | 37712 | 832 | 28 |
| BA1081 | Heidelberg | Turkey Carcass | 5 | 256 | **>160** | 40 | 160 | 80 | 1640 | 7552 | 6312 | 880 | 640 | 1.5 | 2496 | 6304 | 60320 | 37712 | 832 | 28 |
| BA1082 | Heidelberg | Ground Turkey | 4 | 256 | 80 | 40 | 160 | 160 | 1640 | 7552 | 6312 | 880 | 640 | 1.5 | 2496 | 6304 | 120640 | 37712 | 416 | 28 |
| BA1083 | Heidelberg | Turkey Carcass | 10 | 256 | 80 | 40 | 80 | 160 | 1640 | 7552 | 6312 | 880 | 320 | 1.5 | 2496 | 6304 | 60320 | 37712 | 416 | 28 |
| BA1084 | Heidelberg | Young Chicken | 5 | 256 | 80 | 40 | 160 | 160 | 1640 | 7552 | 6312 | 880 | 640 | 1.5 | 2496 | 6304 | 120640 | 37712 | 832 | 56 |
| BA1085 | Heidelberg | Ground Turkey | 6 | 256 | 80 | 40 | 160 | 80 | 1640 | 7552 | 3156 | 880 | 640 | 1.5 | 2496 | 6304 | 60320 | 37712 | 832 | 56 |
| BA1086 | Heidelberg | Ground Turkey | 4 | 256 | 80 | 40 | 160 | 160 | 1640 | 3776 | 6312 | 880 | 640 | 3 | 2496 | 6304 | 120640 | 37712 | 832 | 56 |
| BA1087 | Heidelberg | Ground Turkey | 4 | 256 | 80 | 40 | 160 | 160 | 1640 | 3776 | 6312 | 880 | 640 | 3 | 2496 | 6304 | 120640 | 37712 | 832 | 56 |
| BA1088 | Heidelberg | Young Chicken | 3 | 128 | 80 | 40 | 160 | 160 | 1640 | 3776 | 3156 | 880 | 320 | 1.5 | 2496 | 3152 | 60320 | 37712 | 1664 | 28 |

Shaded values indicating that the growth of isolates were not inhibited at the highest concentration of biocide tested.

Typhimurium* Typhimurium variant O 5 - (Copenhagen)

** Multistate outbreak linked to ground turkey in the United States

MICs in bold indicate the isolate is resistant to the compound with an MIC two-2-fold dilutions above the MIC50 for *Salmonella*

SHB***Clorox brand household bleach is a solution of 5.25% or 52,500 ppm of sodium hypochlorite.
